# Prevalence of common respiratory viruses of infants with respiratory tract infections in European countries in the past decade: a systematic review and meta-analysis comparing between the pre-COVID-19, pandemic, and post-COVID-19 periods

**DOI:** 10.1007/s00431-026-07065-4

**Published:** 2026-05-16

**Authors:** Muhammad Pradhika Mapindra, Muhammad Pradhiki Mahindra, Paul McNamara, Cissy Kartasasmita, Malcolm Gracie Semple, Howard Clark, Jens Madsen

**Affiliations:** 1 Targeted Lung Immunotherapy Group, Neonatology Department, Elizabeth Garrett Anderson Institute for Women’s Health, University College London, 86-96 Chenies Mews, London, UK; 2 Department of Maternal and Fetal Medicine, Elizabeth Garrett Anderson Institute for Women’s Health, University College London, London, UK; 3 University College London Hospital NHS Foundation Trust, London, UK; 4 Harapan Kita National Women and Children Centre, Jakarta, Indonesia; 5 Department of Respiratory Medicine, Liverpool Institute of Child Health and Wellbeing, University of Liverpool, Liverpool, UK; 6 Department of Child Health, Faculty of Medicine, Universitas Padjadjaran, Bandung, Indonesia

**Keywords:** Respiratory infections, Infants, Viruses, Respiratory epidemiology, COVID-19

## Abstract

The purpose of this study is. to determine the leading cause of respiratory tract infections over the decade; this review examined the proportions of airway viruses in infants with respiratory tract infections in Europe before, during, and after the COVID-19 pandemic. The protocol for this systematic review was registered in the PROSPERO database (CRD42024497097). Literature searches in PubMed, Embase, Scopus, and Web of Science (WoS) identified 20,453 studies reporting respiratory viral testing in Europe over the past decade (2012/13–2022/23). Eligible studies were English-language full-text or grey articles with low risk of bias and complete data, and reports that explicitly provided the number of positive airway virus cases (*n*, virus) and the total infant population (*N*). Risk of bias was assessed using the Hoy et al. scoring system. Pooled prevalence estimates (pooled proportion [95% CI]) were calculated for pre-, during-, and post-COVID-19 periods using proportional meta-analysis with the MetaProp package in R. Fifty studies before, ten studies during, and 18 studies after the COVID-19 pandemic were eligible for proportional meta-analyses of viral testing in infants. Before the pandemic, the predominant viruses in infants with respiratory infections were Respiratory Syncytial Virus (RSV) (0.48 [0.41–0.56]), Human rhinovirus (HRV) (0.24 [0.18–0.29]), and Influenza virus (IV) (0.08 [0.04–0.12]). Post-pandemic, the leading viruses were RSV (0.63 [0.51–0.75]), HRV (0.25 [0.11–0.40]), and IV (0.10 [0.00–0.20]) again.

*Conclusions*: RSV and HRV remained the two most identified viruses in infants with respiratory infections, pre- and post-COVID-19 pandemic. Interestingly, both RSV and IV were suppressed during the pandemic. The trajectory dynamics of respiratory viruses were divergent, with resilient viruses (RSV, HRV, and IV) regaining dominance more rapidly than persistently suppressed viruses.

**Table:** 

**What is Known:** • *The COVID-19 pandemic induced an infection precautions policy, which reduced the spread of respiratory infectious diseases.* • *Respiratory Syncytial Virus (RSV) was estimated as one of the most common causes of respiratory infections in infants. Still, there are no reports summarising the geographical distributions of the various viral agents of infant respiratory diseases.* **What is New:** • *This meta-analysis shows that the prevalence of RSV and HRV infections in infants remains high in Europe following the COVID-19 pandemic and the associated precautionary policy. Furthermore, RSV proportionally experienced a marked reduction during COVID-19 pandemic (lockdown periods). However, the immunological debt may have led to a re-emergence in RSV positivity after the lockdown periods.* • *Our proportional meta-analysis indicates the heterogeneous post-pandemic recovery patterns of each respiratory virus commonly identified in infants, where HRV was shown to be resilient whilst RSV was rapidly rebound.*

**Supplementary information:**

The online version contains supplementary material available at 10.1007/s00431-026-07065-4.

## Introduction

Early-life respiratory infections constitute under-five mortality worldwide and nearly 4 million cases of infant deaths [1]. These often precede later-in-life respiratory disorders and may further cause subsequent sub-optimal lung function in adulthood [2–5]. Among pathogens, respiratory viruses are a significant aetiology of early-life respiratory infections [6, 7]. Infants’ susceptibility to virus-laden environments is mainly due to their immunity, which differs from that of adults [4, 6–9].

In the past decade, the emergence of respiratory viruses in Europe has shown a dynamic dependence on seasonality and interactions among viruses [10]. Seasonal patterns and geographical distributions in an area can affect the appearance and peaks of viral infections [11, 12]. Europe is characterised by seasonal and temperature variability throughout the year, with widespread climate change and global warming also affecting seasonal patterns and air temperatures [13]. In the winter of 2019, a novel virus, severe acute respiratory syndrome Coronavirus 2 (SARS-CoV-2), emerged in Wuhan, China, leading to the infectious disease, coronavirus disease 2019 (COVID-19). European countries were, likewise, coping with the COVID-19 pandemic, and the majority of countries resorted to control measures, or nonpharmaceutical interventions (NPIs), to mitigate the virus transmission, such as social distancing, lockdowns, sanitation, and masking [14]. Consequently, such communal habit changes are believed to affect the spread of the pre-existing respiratory viruses, and modified frequencies of these respiratory viruses after the pandemic [15–17]. Investigation into viruses and respiratory infections in infants remains important. No systematic review has compared respiratory virus prevalence in infants throughout the COVID-19 pandemic. This review aims to report the epidemiology of viruses in infants with respiratory infections in Europe, comparing pre-pandemic, pandemic, and post-COVID-19 periods.

## Methods

### Study design

Our systematic review was conducted following the guidelines of Preferred Reporting Items for Systematic Reviews and Meta-Analyses (PRISMA) [18] (Table S1). This systematic review protocol has been registered under the number CRD42024497097 in the PROSPERO database (PROSPERO (york.ac.uk)). This review established the meta-analysis using data from previously published studies; therefore, ethical approval was not required. This systematic review assessed the prevalence of common respiratory viruses underlying respiratory infections in infants across European countries around the pandemic period. The review included infants with respiratory infections and reported viral identification results. Definitions of infancy varied, with some studies grouping infants under 1 year and others under 2 years. The authors of this review agreed to specify infants as those aged 2 years. Comparisons of data were also made across three epochs, before, during, and after the COVID-19 pandemic.

### Eligibility criteria and search strategy

**Fig. 1:**
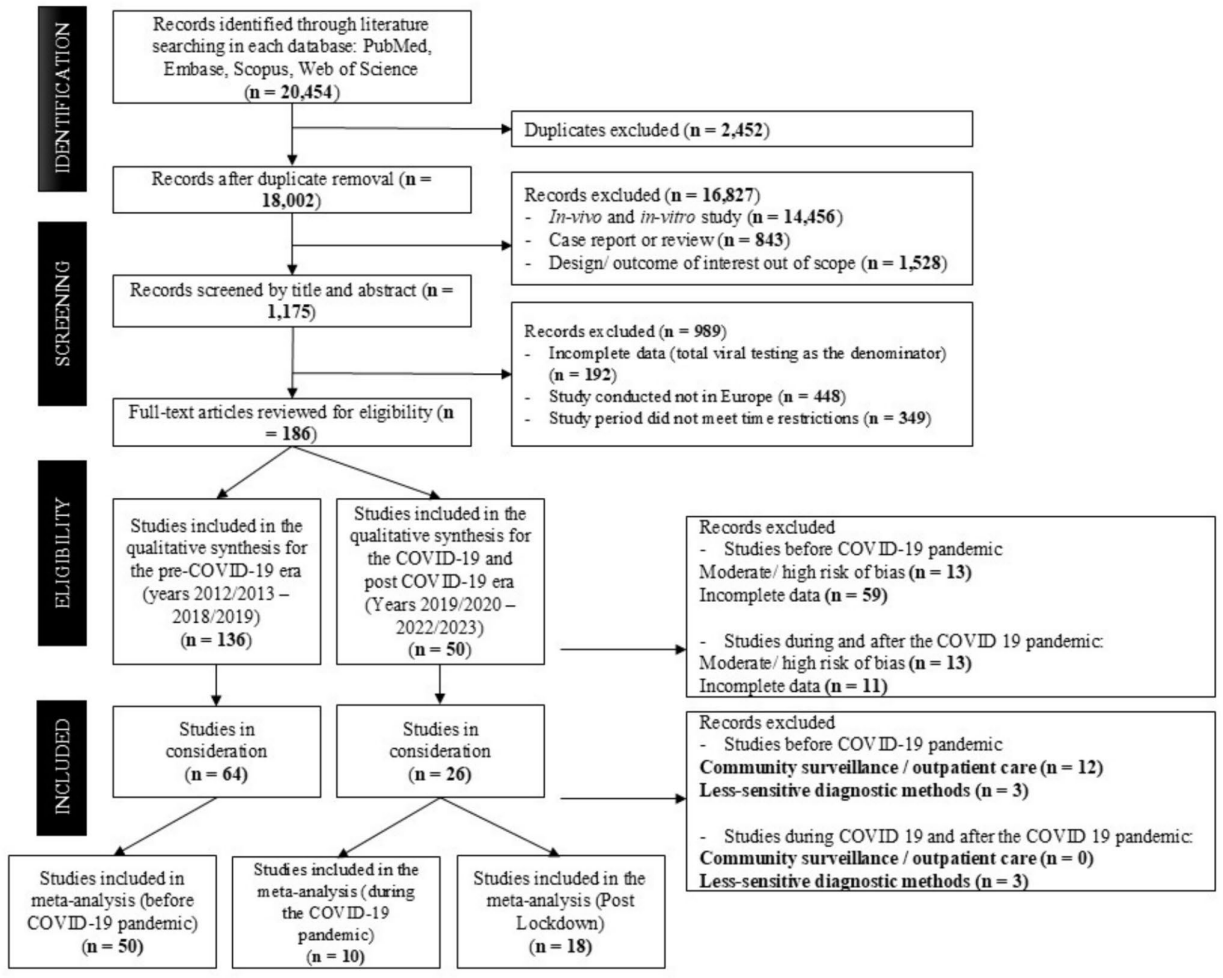
Research flow chart

The literature searches were performed by two review authors (MPA and MPI) independently on the journal databases PubMed, Embase, Scopus, and Web of Science (WoS) with a combination of keywords (Fig. 1). The search strategy consisted of “Infant*” AND “Respiratory Tract Infection*” AND (“Respiratory Syncytial Virus” OR “Rhinovirus” OR “Influenza Virus” OR “Parainfluenzavirus” OR “Bocavirus” OR “Adenovirus” OR “Metapneumovirus”. Language, time, and geographical restrictions were applied to the search strategy. The search was limited to English-language articles from January 2012 to December 2023, including full texts and grey literature on hospitalised infants with complete outcome data. Exclusion criteria included (i) incomplete data, (ii) high bias, (iii) community surveillance, (iv) less-sensitive viral tests like rapid antigen, and (v) participants with pre-existing conditions such as malignancies, HIV, post-surgery, and asymptomatic children. Review papers, preclinical studies, incomplete data, and European respiratory testing outside the region were excluded (Fig. 1).

### Study selection

Two review authors (MPA and MPI) conducted independent searches across the selected databases and identified individual articles based on study titles and abstracts. Study duplications were removed using EndNote, and Rayyan was used to select the studies. We took into account observational studies (including cohort, case–control, or cross-sectional) conducted in European countries that recruited infants below 2 years of age, performed respiratory testing between the years 2012 and 2023, and sufficiently reported the numbers of respiratory testing performed to such age-specific populations of interest (denominators) and the numbers of which tested positive for each virus (nominators). These studies would be further shortlisted if they had sufficiently reported the identification of common respiratory viruses using detection methods with adequate sensitivity and specificity, such as a polymerase chain reaction (PCR) assay, rapid molecular tests, immunofluorescent staining, or PCR-based respiratory panels. Case reports and reviews were excluded. Based on the period of study conduct or the time of viral diagnosis, the study epoch was further divided into the pre-COVID-19 period (2012–2019) and the during/after COVID-19 (2019–2023). Furthermore, due to potential variations between centres in COVID-19 policies, the period during the COVID-19 pandemic referred to the definition from the authors of the included studies, such as “during the COVID-19 era”, whereas the period of the post-COVID-19 pandemic was based on the authors’ definition in their studies, such as “post-lockdown” or “post-COVID-19 pandemic”.

### Data extraction

Data reported in the included studies were independently extracted into a Microsoft Excel spreadsheet by two authors (MPM and MPM). The information retrieved covered: the study identity (authors’ names, publication years), study methods, the study sites (name of the European countries), study epoch (whether before, during, or after the COVID-19), the case definitions, the sampling method and sample type, the viral detection assay (PCR, rapid molecular, rapid antigen, respiratory panels, or immunofluorescent staining), number of total samples tested for each virus, and number of positives for each virus. All disagreements between the review authors were resolved by further discussion and consultations with the senior authors (JM and HW).

### Risk of bias assessments

The Hoy et al. assessment tools (Table S3) were used qualitatively to assess the risk of bias for each included study. The assessments were estimated as low (7–10), moderate (4–6), and high (0–3) [19].

### Data analysis

The overall results were visualised using forest plots and summary tables. The prevalence of each respiratory virus was estimated using the study weights. The heterogeneity of the included observational studies was assessed using the Cochran Q test and the I^2^ statistic [20]. The prevalence of the respective viruses was estimated using a random-effects meta-analysis, given heterogeneity across studies, with a random-effects model applied to studies with an I^2^ > 40%. *P*-values < 0.05 were considered statistically significant. The meta-analyses for prevalence in this study were conducted using the “metaprop” function from the meta package (version 6.5–0) in R (version 4.3.1). Moreover, bubble plots on the Europe map were also generated under the packages “ggplot” and “rnaturalearth” [21, 22].

## Results

### Study characteristics

The literature searches yielded 20,454 studies, and 2452 duplicates were removed. A further 186 studies were selected from the remaining 1175 that underwent title and abstract screening. Following the exclusion of publications due to multiple factors (Fig. 1), 136 studies with viral testing performed before the COVID-19 pandemic [23–153], and 50 studies [24–26, 29, 32, 33, 41, 43, 50, 52, 53, 57, 62, 67, 74, 78, 154–187] during/after the COVID-19 pandemic were considered further for qualitative risk of bias assessments. Only those studies with low bias scores (7–10) (Table S2 & S3), respiratory testing performed within the 2012/2013–2022/23 window, sampling performed in infants below 2 years of age, enrolling hospitalised infants, and employing appropriate viral diagnostic methods, were considered eligible for further meta-analyses. Community surveillance studies and studies enrolling outpatient children were removed from the pre-pandemic group (12 studies) (Fig. 1) [23, 30, 51, 58, 73, 76, 99, 102, 137, 138, 141, 144]. Studies employing rapid antigen tests or unclear viral identification methods were excluded from meta-analyses, amounting to three studies for the period before the COVID-19 pandemic [60, 76, 147] and three studies during/after the pandemic period [24, 53, 169].

The bias assessments resulted in a total number of 64 studies with viral diagnoses before the COVID-19 pandemic in Europe [25, 27, 28, 32, 33, 36, 42, 44, 49, 52, 54, 63, 64, 66, 68, 69, 78, 80–86, 89, 91, 95, 100, 103, 104, 107, 108, 110–113, 116, 117, 124–126, 128, 131, 143, 149, 165, 176, 188, 189] and 26 papers for the period during/after the COVID-19 pandemic [25, 32, 33, 52, 78, 155, 158, 159, 161–165, 167, 168, 173, 175–177, 179–184, 187]. Furthermore, the period during/after COVID-19 was classified into during the COVID-19 pandemic (10 studies) [32, 33, 78, 159, 167, 177, 179, 181, 184, 187], and after the COVID-19 pandemic or post-lockdown period (18 studies) [25, 33, 63, 155, 158, 159, 161–165, 168, 169, 173, 175, 176, 182, 183] (Fig. 1). The respiratory viruses considered in the current systematic review were respiratory syncytial virus (RSV), human rhinovirus (HRV), adenovirus (ADV), influenza virus (IV), bocavirus (BoV), parainfluenzavirus (PIV), metapneumovirus (MPV), and human coronavirus (hCOV). RSV testing was reported by 90.00% of the included studies that performed viral testing before the global pandemic and by all included studies that performed viral testing during and after the COVID-19 pandemic. Furthermore, HRV was the second-most frequently tested virus over the decade (2012/13–2022/23). Conversely, BoV was the least tested virus before the COVID-19 pandemic (38.00%), whereas IV was the least tested during the pandemic (40.00%) and after the pandemic (22.22%) (Table 1).

**Table 1:** Characteristics of the included studies

Study characteristics	Pre-COVID-19 (*N* = 50)	During COVID-19 (*N* = 10)	Post-COVID-19 (*N* = 18)
Age range			
• < 1 years of age • < 2 years of age	24 (48.00%) 50 (100.00%)	5 (50.00%) 10 (100.00%)	8 (44.44%) 18(100.00%)
Diagnosis			
• Lower respiratory tract infections (LRTIs): • Other than LRTIs	29 (58.00%) 21 (42.00%)	6 (60.00%) 4 (40.00%)	14 (77.78%) 4 (22.22%)
Design of studies			
• Case–control • Cohort • Cross-sectional	3 (6%) 44 (88.00%) 3 (6%)	0 (0.00%) 8 (80.00%) 2 (20.00%)	0 (0.00%) 16 (88.89%) 2 (11.11%)
Numbers of studies with viral testings			
• Respiratory Syncytial Virus (RSV) • Human Rhinovirus (hRV) • Human Metapneumovirus (hMPV) • Adenovirus • Human Bocavirus (hBOV) • Influenza virus • Parainfluenzavirus • Human Coronavirus (hCOV)	45 (90.00%) 33 (66.00%) 24 (48.00%) 20 (40.00%) 19 (38.00%) 26 (52.00%) 23 (46.00%) 21 (42.00%)	10 (100.00%) 8 (80.00%) 6 (60.00%) 6 (60.00%) 5 (50.00%) 4 (40.00%) 6 (60.00%) 6 (60.00%)	18 (100.00%) 12 (66.67%) 10 (55.56%) 9 (50.00%) 6 (33.33%) 4 (22.22%) 10 (55.56%) 5 (27.78%)

### Prevalence of respiratory viruses before the COVID-19 pandemic (from the Year 2012/2013 to the COVID-19 pandemic)

The pre-pandemic meta-analysis showed that RSV was the leading viral aetiology of infants with respiratory tract infections (0.48; 95% CI [0.41–0.56]) (Figure S1, Table 2). Other tested viruses by pooled prevalence, in declining order, were as follows: HRV (0.24; 95% CI [0.18–0.29]) (Figure S2, Table 2), influenza virus (IV) (0.08; 95% CI [0.04–0.12]) (Figure S3, Table 2), and adenovirus (ADV) (0.07; 95% CI [0.05–0.10]) (Figure S4, Table 2). Three viruses share similar pooled prevalence: BoV (0.06; 95% CI [0.04–0.07]), parainfluenzavirus (PIV) (0.06; 95% CI [0.04–0.07]), and human coronavirus (hCOV) (0.06; 95% CI [0.04–0.07]) (Figure S5-S7, Table 2). Human metapneumovirus (hMPV) was the lowest detected, at 0.05 (95% CI [0.04–0.06]) (Figure S8,Table 2). Before COVID-19, European reports on infants with respiratory infections showed that most positive cases were for RSV and HRV (Figure S9).

**Table 2:** Prevalence of common respiratory viruses in infants tested before COVID-19 pandemic, sorted from the highest to the lowest by pooled proportions

Virus	Number of studies	Pooled prevalence	95% CI
Respiratory syncytial virus (RSV)	45	0.48	0.41–0.56
Human rhinovirus (hRV)	33	0.24	0.18–0.29
Influenza virus (IV)	26	0.08	0.04–0.12
Adenovirus (ADV)	20	0.07	0.05–0.10
Bocavirus (BoV)	19	0.06	0.04–0.07
Parainfluenzavirus (PIV)	23	0.06	0.04–0.07
Human coronavirus (hCOV)	21	0.06	0.04–0.07
Human metapneumovirus (hMPV)	24	0.05	0.04–0.06

### Prevalence of respiratory viruses during the COVID-19 pandemic

Based on our meta-analysis of the included studies that performed respiratory viral testing during the COVID-19 pandemic, HRV, with a pooled prevalence of 0.45 (95% CI [0.21; 0.68]) (Figure S10, Table 3), was the most prevalent virus detected. By a decreasing sequence of the pooled proportions, there were also changes in the prevalence ranking for other viruses: RSV (0.15; 95% CI [0.00; 0.34]) (Figure S11, Table 3), ADV (0.09; 95% CI [0.03; 0.15]) (Figure S12, Table 3), hCOV (0.08; 95% CI [0.01; 0.14]) (Figure S13, Table 3), hMPV (0.06; 95% CI [0.00; 0.14]) (Figure S14, Table 3), BoV (0.03; 95% CI [0.01; 0.05]), and PIV (0.02; 95% CI [0.00; 0.05]) (Figure S15 – S16, Table 3). Surprisingly, IV appeared to be absent in this period (0.00; 95% CI [0.00; 0.00]) (Figure S17, Table 3). The distribution map revealed that HRV-positive cases stayed high along the COVID-19 pandemic, whereas RSV-positive cases decreased (Figure S18).

**Table 3:** Prevalence of common respiratory viruses in infants tested during COVID-19 pandemic, sorted from the highest to the lowest by pooled proportions

Virus	Number of studies	Pooled prevalence	95% CI
Human rhinovirus (hRV)	8	0.45	0.21–0.68
Respiratory syncytial virus (RSV)	10	0.15	0.00–0.34
Adenovirus (ADV)	6	0.09	0.03–0.15
Human coronavirus (hCOV)	6	0.08	0.01–0.14
Human metapneumovirus (hMPV)	6	0.06	0.00–0.14
Bocavirus (BoV)	5	0.03	0.01–0.05
Parainfluenzavirus (PIV)	6	0.02	0.00–0.05
Influenza virus (IV)	4	0.00	0.00–0.00

### Prevalence of respiratory viruses post-pandemic period of COVID-19

For the meta-analysis of the included studies that performed respiratory viral testing post-COVID-19 pandemic, RSV resurged as the most prominent virus detected in infants, with a pooled prevalence of 0.63; 95% CI [0.51; 0.75]) (Figure S19, Table 4). Meanwhile, HRV (0.25; 95% CI [0.11; 0.40]) ranked second (Figure S20, Table 4). The prevalence of IV rose to the third highest (0.10; 95% CI [0.00; 0.20]) (Figure S21, Table 4), followed by hCOV (0.06; 95% CI [0.02; 0.09]) (Figure S22, Table 4). Moreover, hMPV (0.04; 95% CI [0.02; 0.07]), PIV (0.04; 95% CI [0.03; 0.06]), and BoV (0.04; 95% CI [0.03; 0.05]) showed an equivalent pooled prevalence (Figure S23–S25, Table 4). Moreover, ADV had the lowest pooled prevalence (0.03; 95% CI [0.02; 0.05]) (Figure S26, Table 4). The map showcases that RSV, HRV, and IV became the predominant respiratory pathogens in infants after the COVID-19 pandemic, with the pooled prevalence of RSV and IV showing a noticeable resurgence (Figure S27).

**Table 4:** Prevalence of common respiratory viruses in infants tested after COVID-19 pandemic, sorted from the highest to the lowest by pooled proportions

Virus	Number of studies	Pooled prevalence	95% CI
Respiratory syncytial virus (RSV)	18	0.63	0.51–0.75
Human rhinovirus (hRV)	12	0.25	0.11–0.40
Influenza virus (IV)	4	0.10	0.00–0.20
Human coronavirus (hCOV)	5	0.06	0.02–0.09
Human metapneumovirus (hMPV)	10	0.04	0.02–0.07
Parainfluenzavirus (PIV)	10	0.04	0.03–0.06
Bocavirus (BoV)	6	0.04	0.03–0.05
Adenovirus (ADV)	9	0.03	0.02–0.05

## Discussion

Our meta-analysis indicated that RSV and HRV were the two most prevalent viral causes of respiratory infections among infants in Europe over the decade from 2012/13 to 2023, across the epochs before, during, and after the COVID-19 pandemic. Previous studies also showed RSV underlay respiratory infections in children worldwide, regardless of age levels [190–192]. Other studies from Europe conducted during a period earlier than this systematic review found that either RSV [193] or HRV [194] was primarily identified viruses in respiratory samples of infants. Outside Europe, either RSV or HRV was also reportedly the predominant virus isolated from infants’ respiratory tracts [195–198]. According to our meta-analyses, the proportion of HRV remained prominent, while RSV dwindled, during the COVID-19 pandemic and NPI implementation. The lowest-detected virus shifted from hMPV in the pre-pandemic period to IV during the COVID-19 emergence, and to ADV after the pandemic (Tables 2, 3, and 4). Additionally, IV ranked third and, similar to RSV, declined proportionally during the COVID-19 pandemic (Table 3). Other studies also documented that the circulation of some respiratory viruses, including RSV, dropped following pandemic-driven NPIs [15–17]. The NPI policy implementation seems responsible for reduced transmission of respiratory viruses broadly, and the shift of respiratory viruses potentially indicates differential virus sensitivity to such NPIs [199, 200]. The marked reduction in RSV and the near-complete disappearance of IV at the onset of the COVID-19 pandemic, observed in our meta-analysis, support global reports of suppression of enveloped respiratory viruses following NPI implementation. Enveloped viruses, including RSV and IV, possess a lipid-composed envelope that renders them susceptible to sanitisation and environmental disruption [199, 201, 202]. Contrastingly, HRV is a non-enveloped virus in nature, rendering the virus more environmentally stable and less affected by alcohol-based disinfectants, an element of routine sanitisation during NPI implementation [203, 204]. This factor may explain the continued circulation of HRV despite the pandemic-related NPIs.

Our proportional meta-analysis demonstrates divergent post-pandemic recovery trajectories across respiratory viruses, rather than a uniform rebound pattern. It showed a relative increase in the proportion of infants testing positive for RSV, IV, PIV, and BoV after the COVID-19 pandemic. Notably, RSV rapidly regained significant prominence as the leading cause of viral-associated respiratory infections in infants in Europe, followed by RV as the second (Tables 3 and 4). Despite a pandemic-driven drop, both RSV and IV showed a rebound pattern after the COVID-19 period and the loosening of NPI policies [205–207], suggesting that the restoration of social mixing may differentially influence viral transmission dynamics. Post-pandemic viral resurgences also suggest that certain viruses may require the re-establishment of dense contact networks before transmission intensity recovers. These fluctuations likely reflect the combined effects of NPI, altered social mixing patterns, and the “immunity debt” phenomenon. Reduced viral exposure during lockdown periods within the COVID-19 pandemic peak, particularly for RSV, might cause the accumulation of susceptible infants and potentially reduced exposure to maternal antibodies, thereby contributing to the intensified post-pandemic resurgence [208–210]. Despite the effectiveness of NPIs in limiting the person-to-person spread of respiratory viruses, inadequate transfer of maternally derived passive immunity may promote susceptibility of babies to these viruses. During the pandemic, breastfeeding, which provides infants with passive immunity against respiratory viruses, became infrequent [211]. Meanwhile, reduced exposure of childbearing women to respiratory viruses could deprive infants of specific maternal-derived antibodies transferred in utero via the placenta. Collectively, these might explain the increased detection rates of RSV and IV upon NPI loosening after the COVID-19 pandemic, given that some factors occurring during the pandemic may have driven the rise in detected viral prevalence post-pandemic. Altered RSV epidemiological patterns encourage the need for adaptive timing of RSV prophylaxis strategies, including maternal vaccination and monoclonal antibodies.

A decreasing trend was observed in ADV, hMPV, hCOV, and PIV. Following the COVID-19 pandemic, the number of hospitalised children who tested positive for hMPV, PIV, and BoV individually fell below hCOV (Tables 2, 3, and 4). Viral interference and ecological competition during periods of high SARS-COV-2 circulation may have influenced the temporal redistribution of respiratory viruses [209]. Subsequently, the post-pandemic period appears to represent an ecological restructuring of the respiratory viral landscape, where NPIs may have disrupted the equilibrium of respiratory virus circulation [212–214]. The rapid resurgences suggest that both RSV and HRV are epidemiologically resilient viruses with high transmissibility within susceptible infant populations, and that incomplete immunity after infection may occur [215–217]. These high-resilience viruses seemed to reoccupy ecological niches rapidly, whereas persistently suppressed viruses failed to regain pre-pandemic circulation intensity. Ecological competition and viral interference mechanisms may have favoured the resurgence of highly transmissible pathogens (RSV and HRV), limiting the re-establishment of other respiratory viruses in the post-pandemic period [215, 218]. When these two dominant viruses are present, they may occupy ecological niches, limit the co-circulation of other, less dominant respiratory viruses, and reduce opportunities for secondary viral infections [215, 218, 219]. Compared with the resilient viruses, both PIV and hMPV appear susceptible to NPIs and therefore have lower baseline transmissibility. Besides, both exhibit strong seasonality and rely heavily on close-contact networks, which may be effectively suppressed by social distancing [214, 220]. The tedious alteration in hCOV prevalence is likely attributable to the enhanced diagnostic focus on coronaviruses following the emergence of SARS-CoV-2, along with broader use of multiplex respiratory panels, likely increasing the detection of hCOV [221]. In addition, hCOV may have been less susceptible to suppression by NPIs than paramyxoviruses, which rely on close-contact transmission and exhibit pronounced seasonality.

## Limitations

This systematic review excluded non-English studies, thus introducing publication bias. The search strategy, limited to 2023, underrepresents high-quality studies from 2024 that could offer more post-pandemic insights. Variations in study methods and outcomes likely caused heterogeneity bias, with heterogeneity ranging from 80 to 100%, indicating diverse populations. As a result, findings may have limited applicability despite using random-effects models. Most studies were observational, risking bias from confounders such as healthcare differences across Europe, RSV season severity, diagnostic methods, testing access and techniques, and pre-existing conditions.

## Conclusions

In Europe, RSV and HRV remained the most common viruses in infants with respiratory infections, before and after COVID-19. The suppression of enveloped viruses such as RSV was observed at the pandemic onset when NPIs existed. The sharp RSV resurgence advocates prophylaxis strategies. The pandemic impact on different viral species underscores the role of viral structure in transmission and shows how behavioural interventions shape respiratory virus epidemiology. Our findings support the hypothesis of an ecological restructuring of the respiratory virome, whereby NPI disrupted pre-existing viral hierarchies and eventually allowed certain highly transmissible or resilient pathogens to regain dominance. Since our study is limited to Europe, further research is needed elsewhere to understand recent changes in circulating respiratory viruses.

## Supplementary information

Below is the link to the electronic supplementary material.

ESM 1 (DOCX 24.2 KB)

ESM 2 (XLSX 16.4 KB)

ESM 3 (PDF 91.1 KB)

ESM 4 (DOCX 7.18 MB)

ESM 5 (DOCX 61.2 KB)

## Data Availability

The supplementary data for this study are available upon request from the corresponding author.

## Abbreviations

ADV: Adenovirus
BoV: Bocavirus
CI: Confidence interval
COVID-19: Coronavirus disease 2019
hCOV: Human coronavirus
HRV: Human rhinovirus
IV: Influenza virus
MPV: Metapneumoniavirus
PCR: Polymerase chain reaction
PIV: Parainfluenza virus
PRISMA: Preferred Reporting Items for Systematic Reviews and Meta-Analyses
RSV: Respiratory syncytial virus
WoS: Web of science
