# Supplementary material for: Prevalence of common respiratory viruses of infants with respiratory tract infections in European countries in the past decade: a systematic review and meta-analysis comparing between the pre-COVID-19, pandemic, and post-COVID-19 periods
Source: Eur J Pediatr. 2026 May 16;185(6):401. doi: 10.1007/s00431-026-07065-4 (PMC13179882; doi:10.1007/s00431-026-07065-4)
Supplement: Supplementary file 4 — (DOCX 7.18 MB) [file 431_2026_7065_MOESM4_ESM.docx]

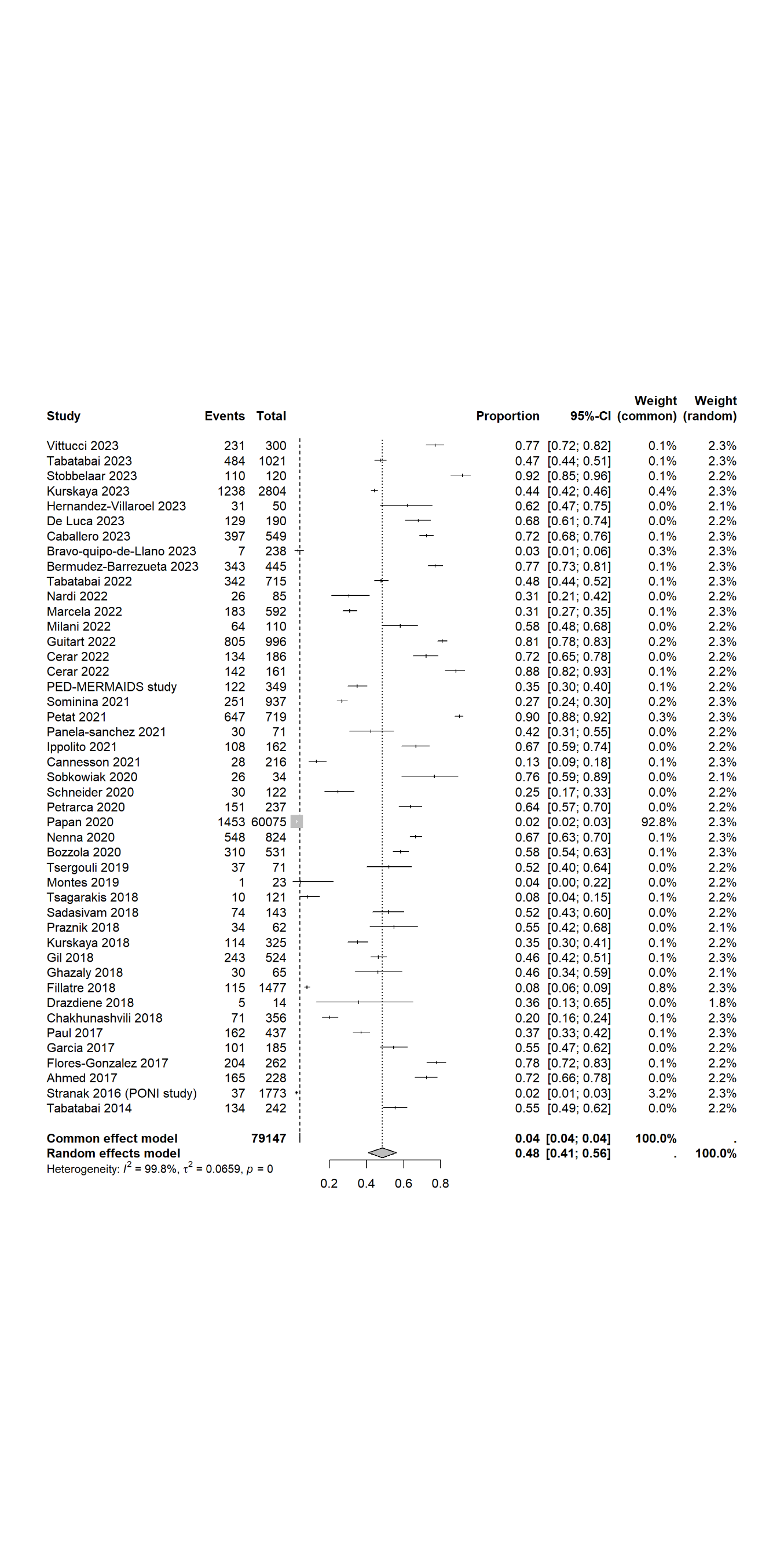


**Figure S1.** Forest Plot of Proportion of Respiratory Syncytial Virus (RSV) before COVID-19


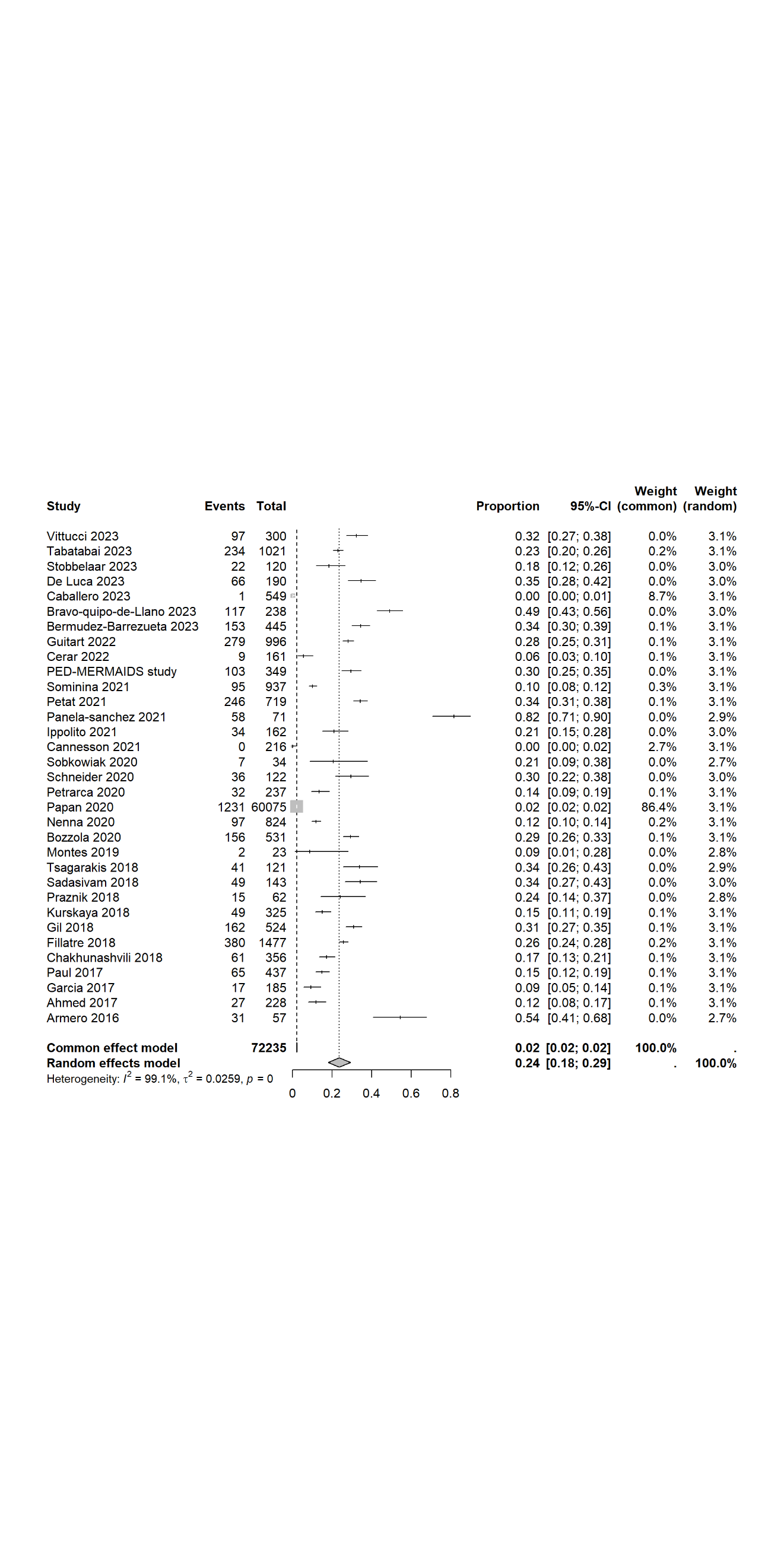


**Figure S2.** Forest Plot of Proportion of Human Rhinovirus (HRV) before COVID-19


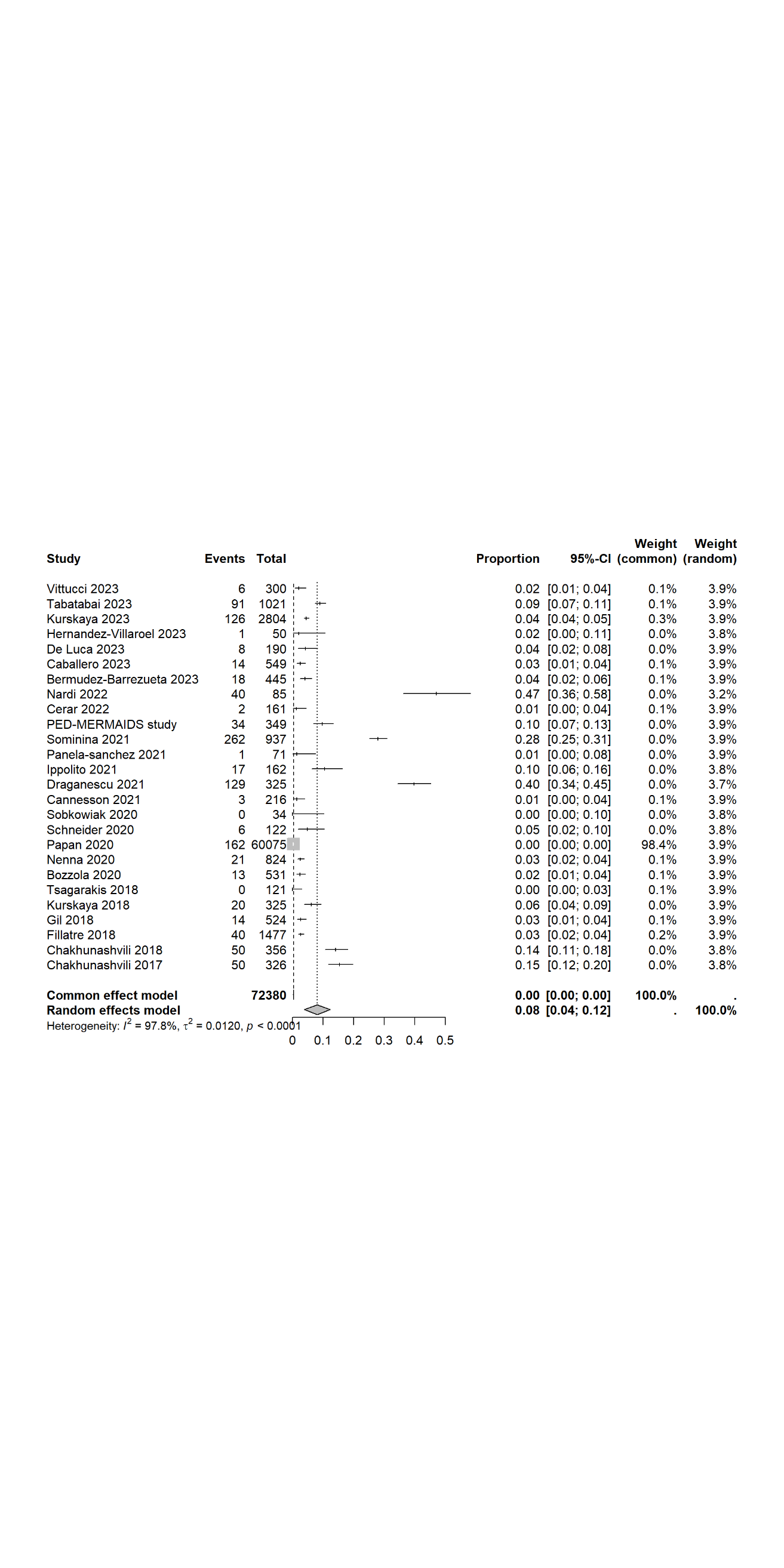


**Figure S3.** Forest Plot of Proportion of Influenza Virus (IV) before COVID-19


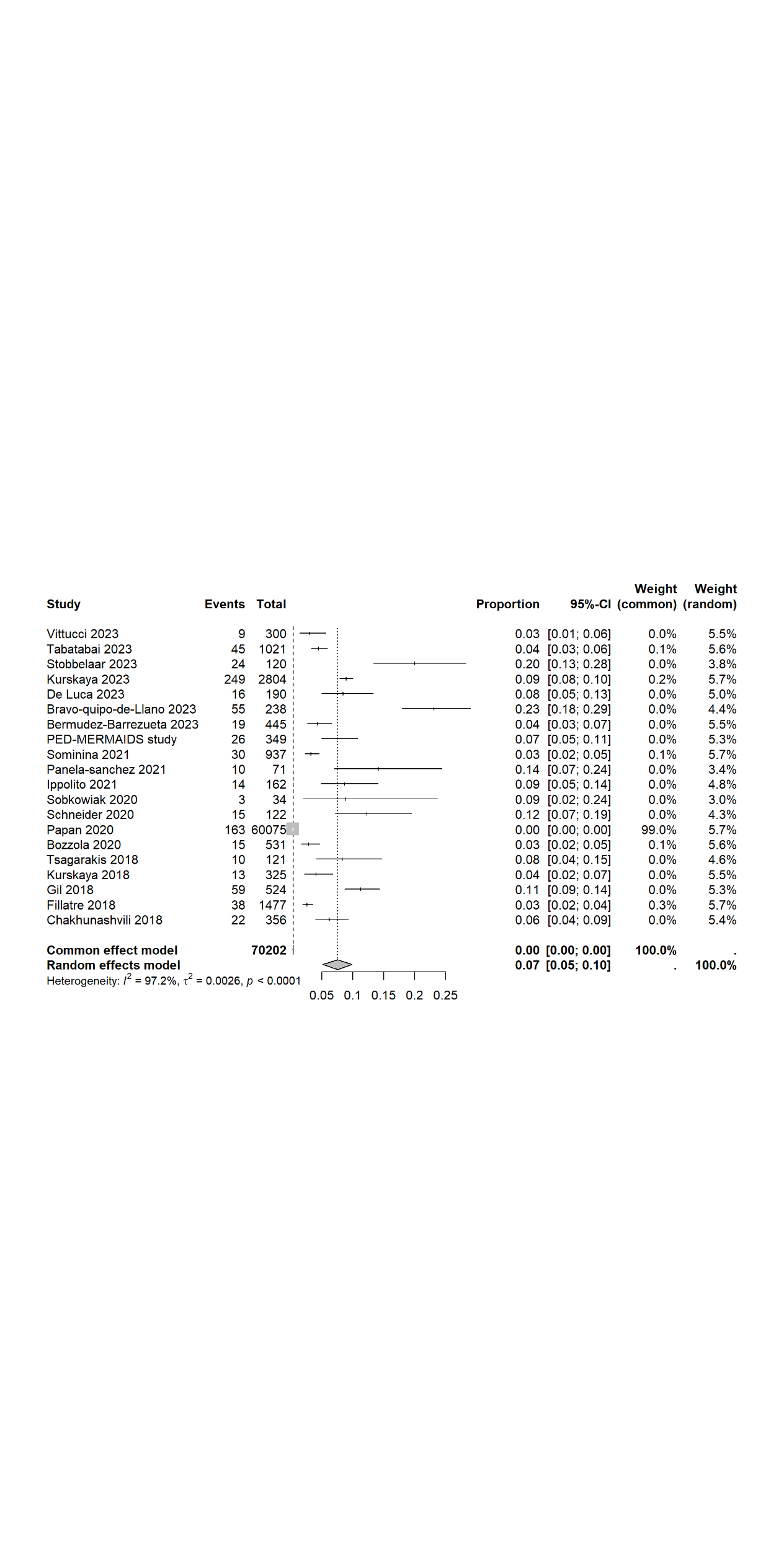


**Figure S4.** Forest Plot of Proportion of Adenovirus (ADV) before COVID-19


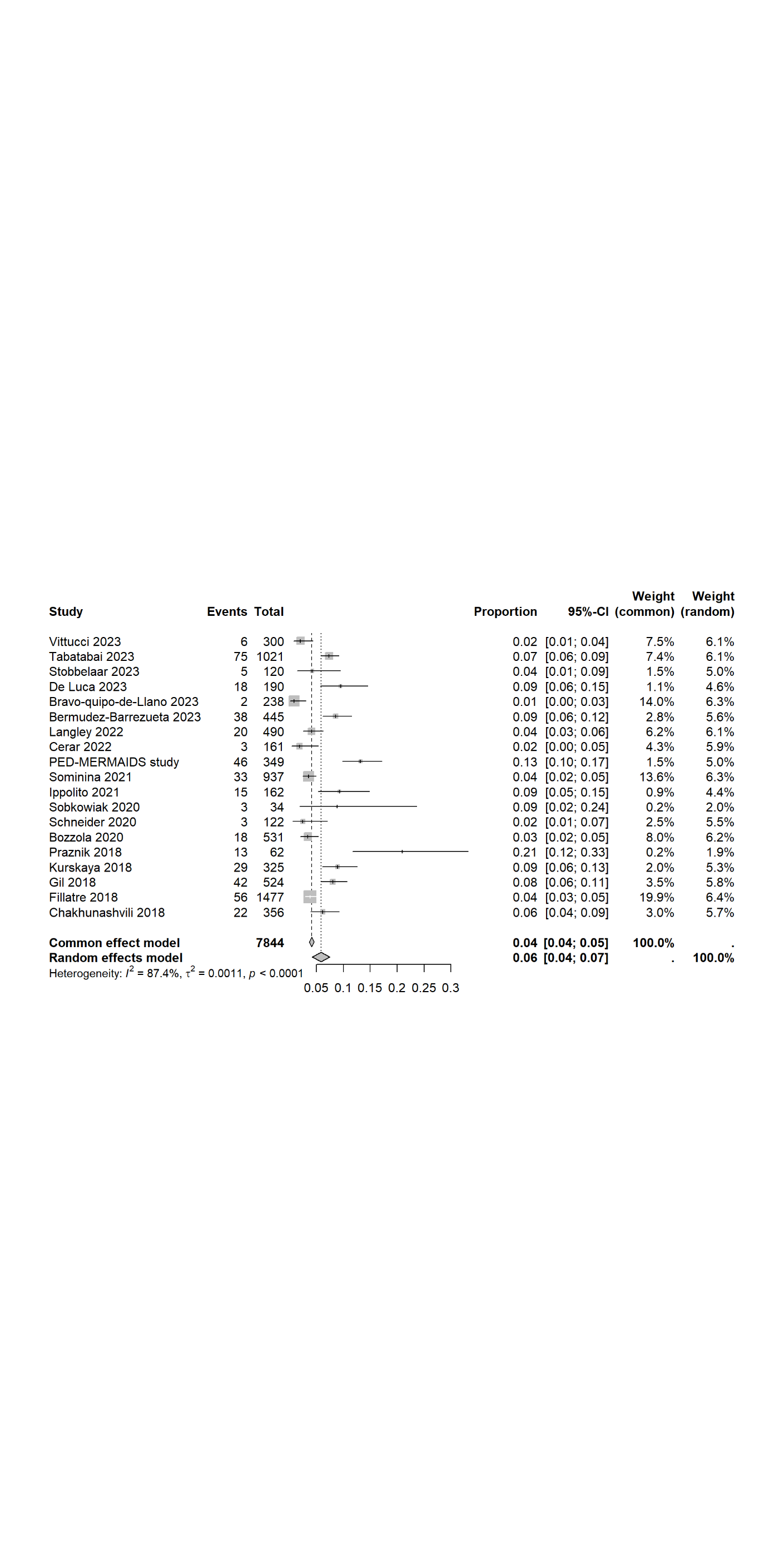


**Figure S5.** Forest Plot of Proportion of Bocavirus (BoV) before COVID-19


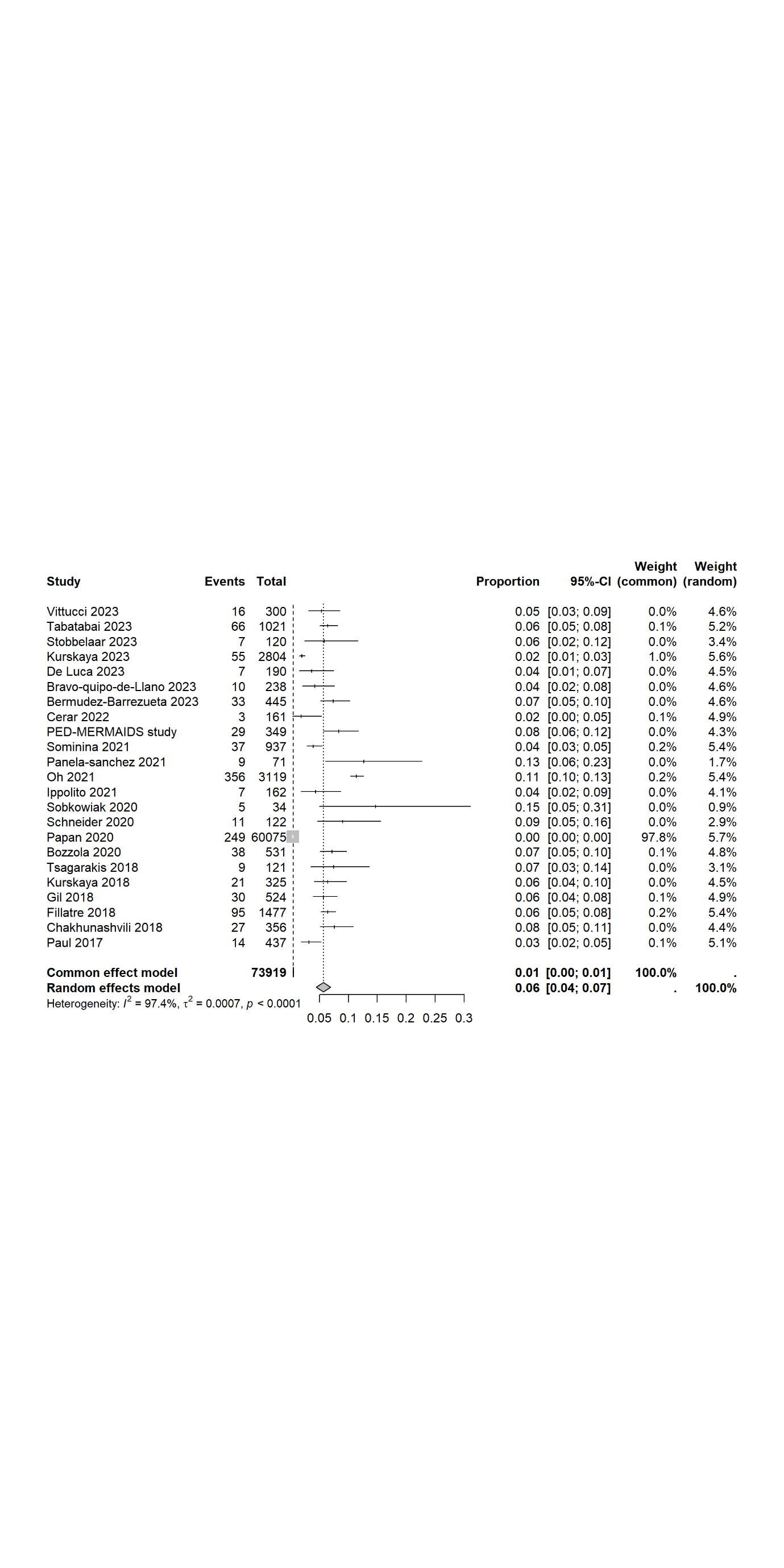


**Figure S6.** Forest Plot of Parainfluenzavirus (PIV) before COVID-19


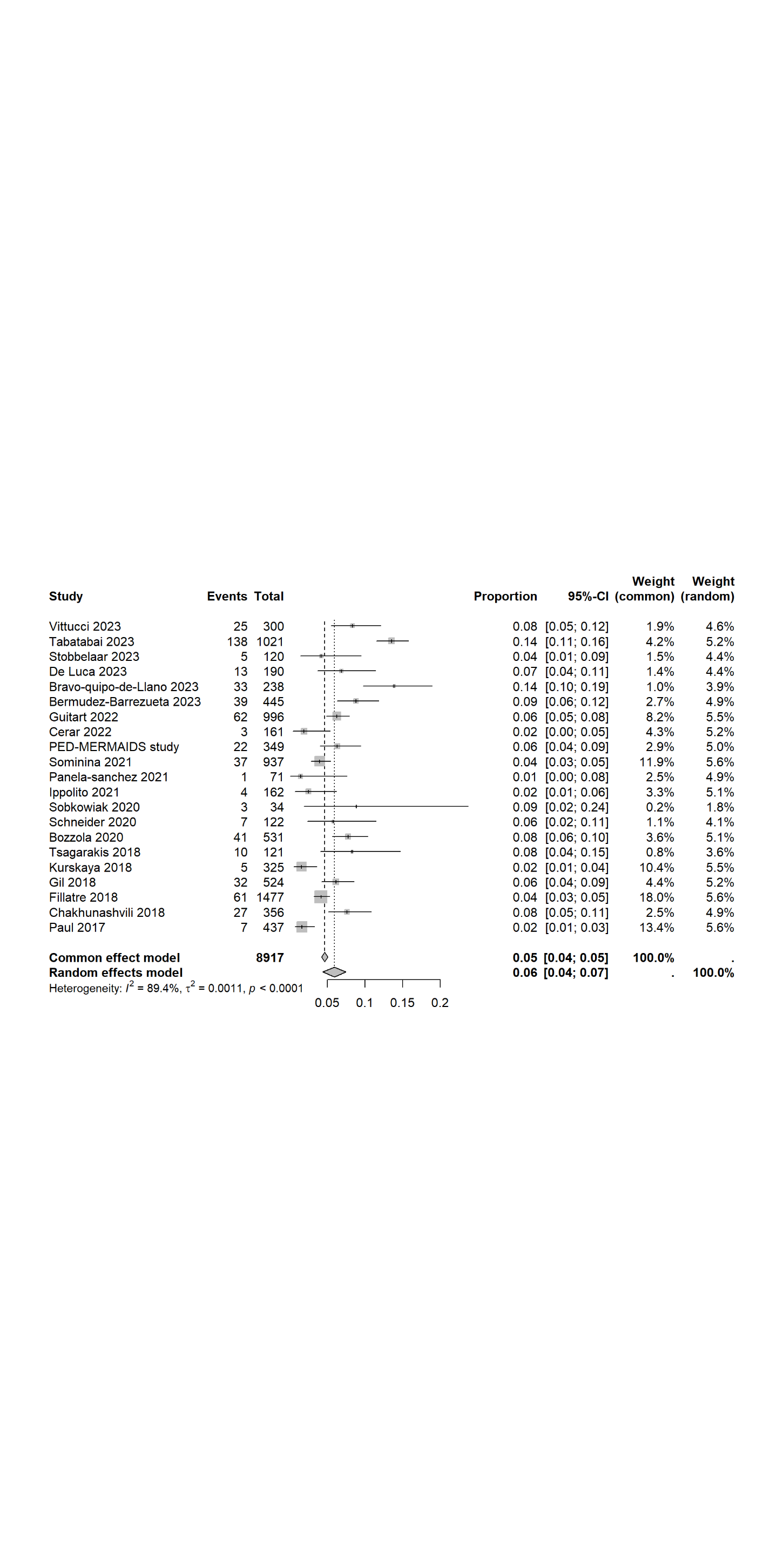


**Figure S7.** Forest Plot of human Coronavirus (hCOV) before COVID-19


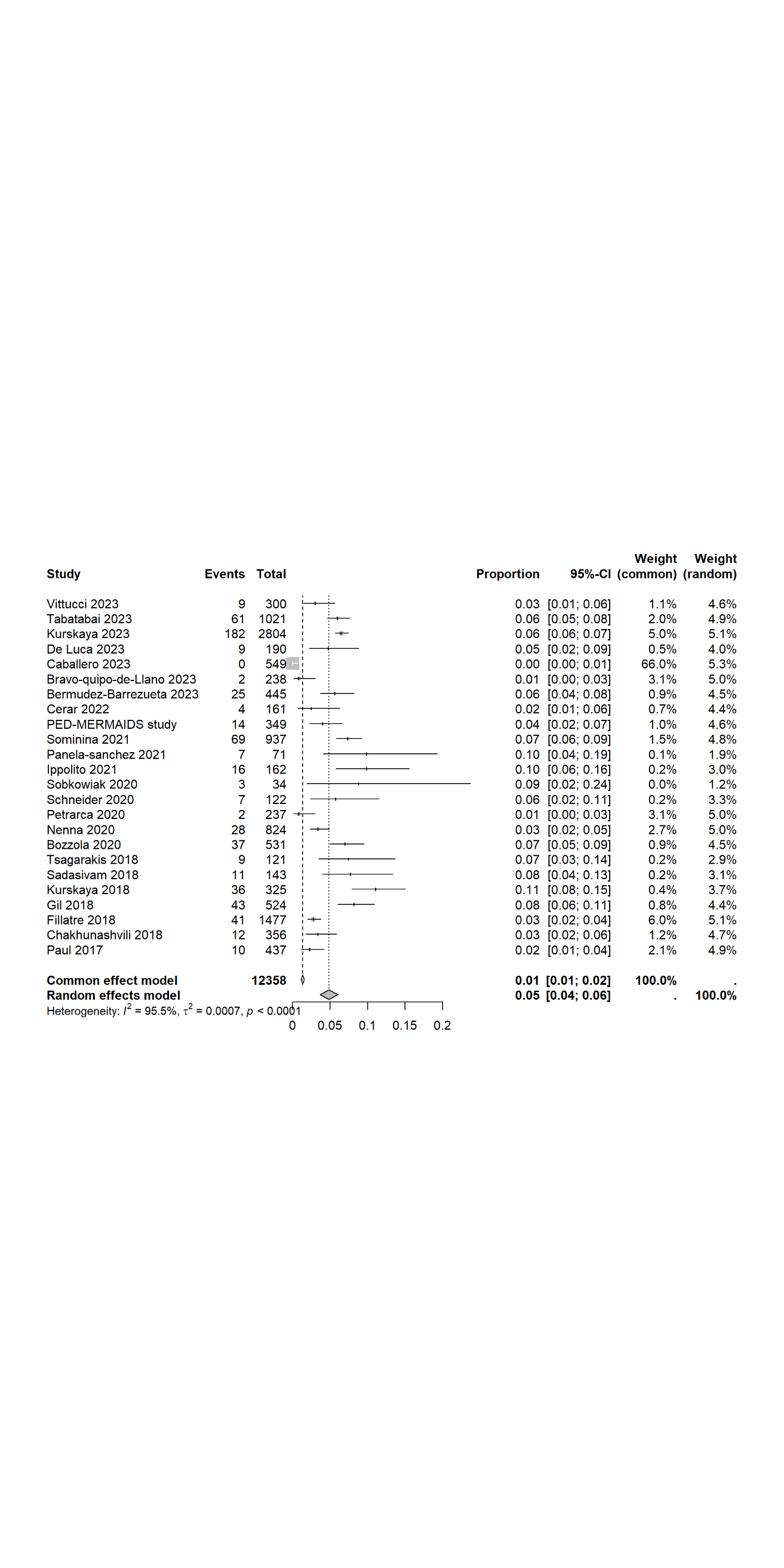


**Figure S8.** Forest Plot of Human Metapneumovirus (hMPV) before COVID-19


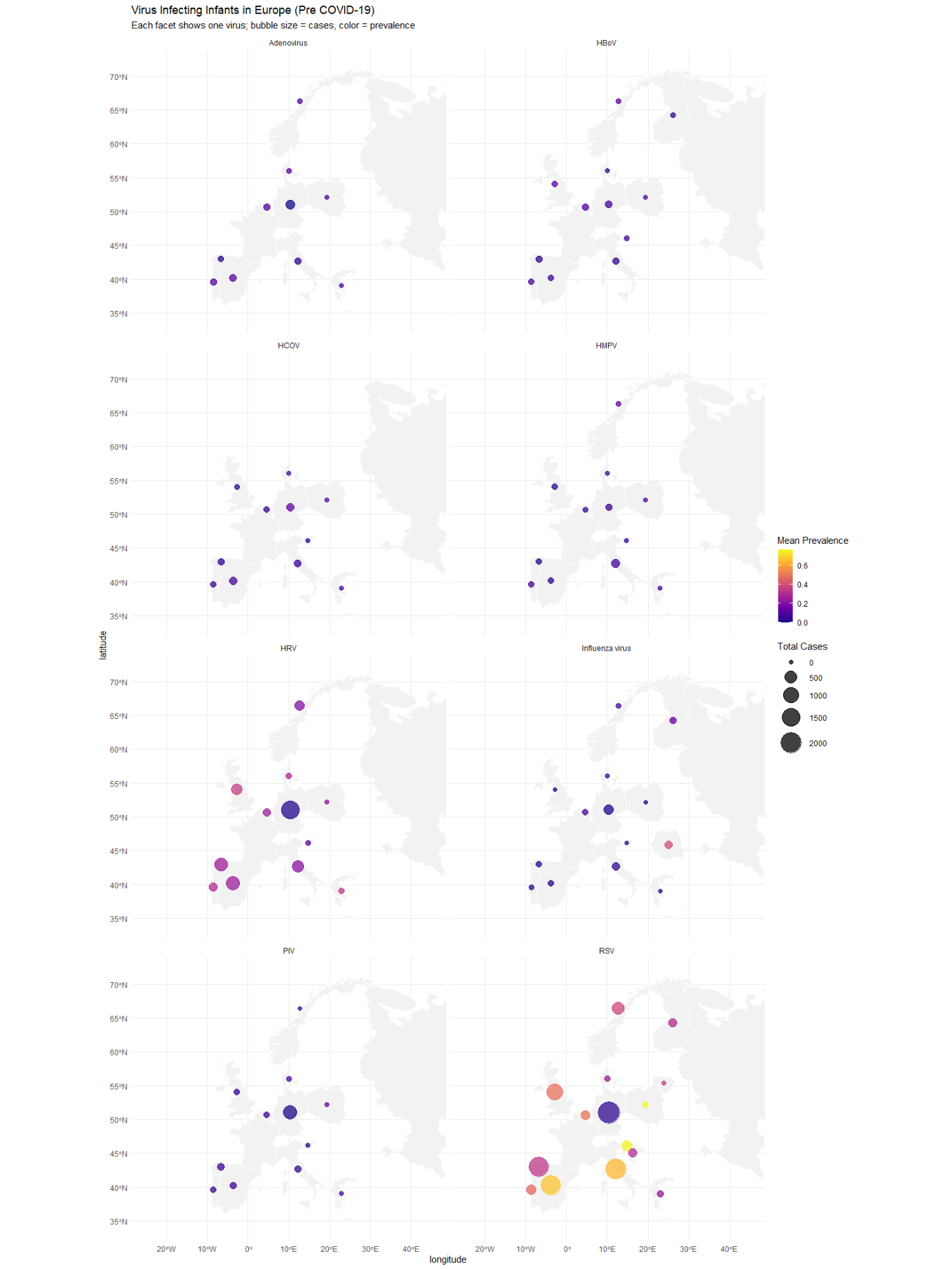


**Figure S9.** Bubble plots for prevalence of viral identifications in infants with respiratory tract infection in European countries prior to COVID-19 pandemic.


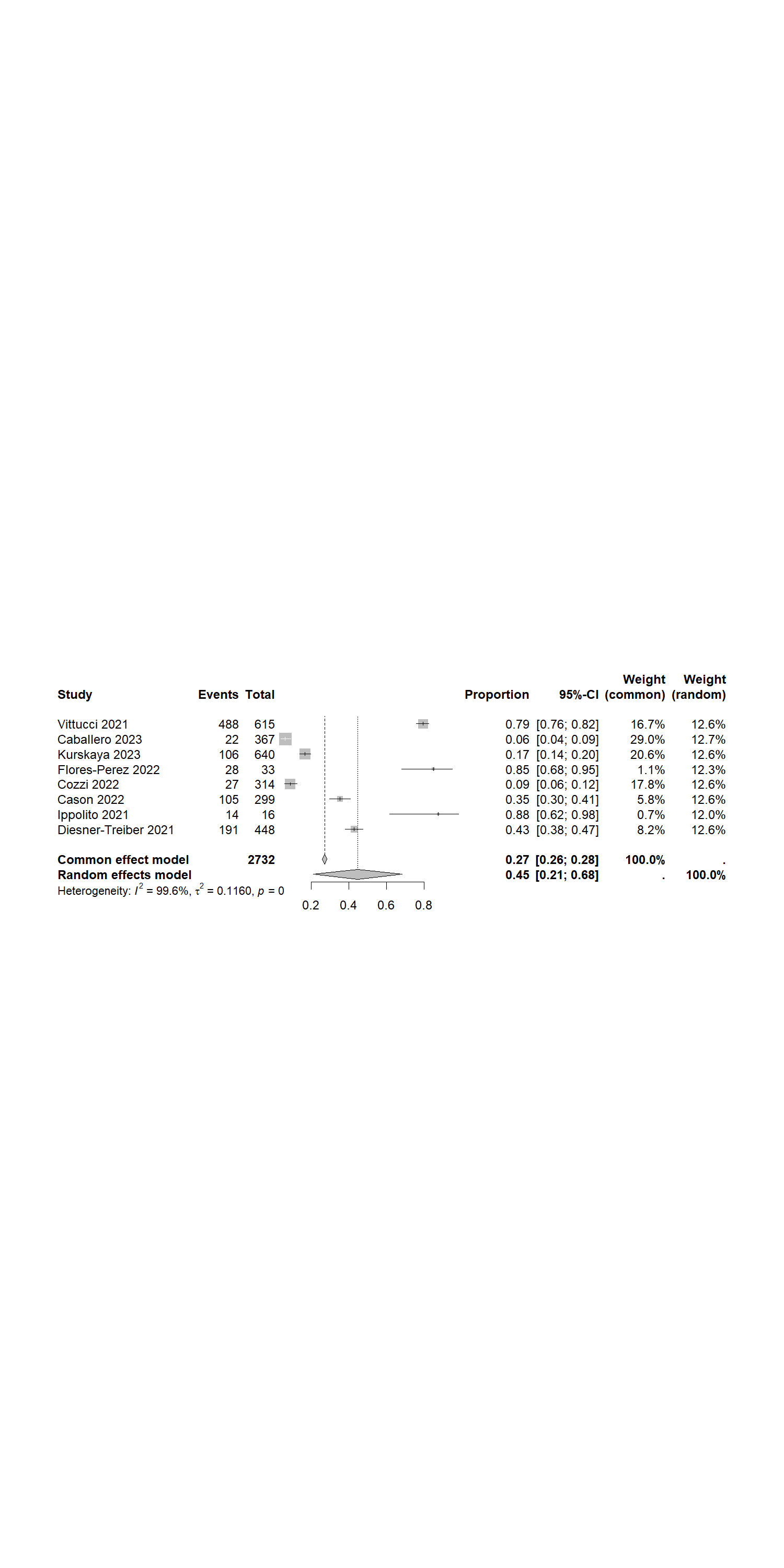


**Figure S10.** Forest Plot of Proportion of Human Rhinovirus (HRV) during COVID-19


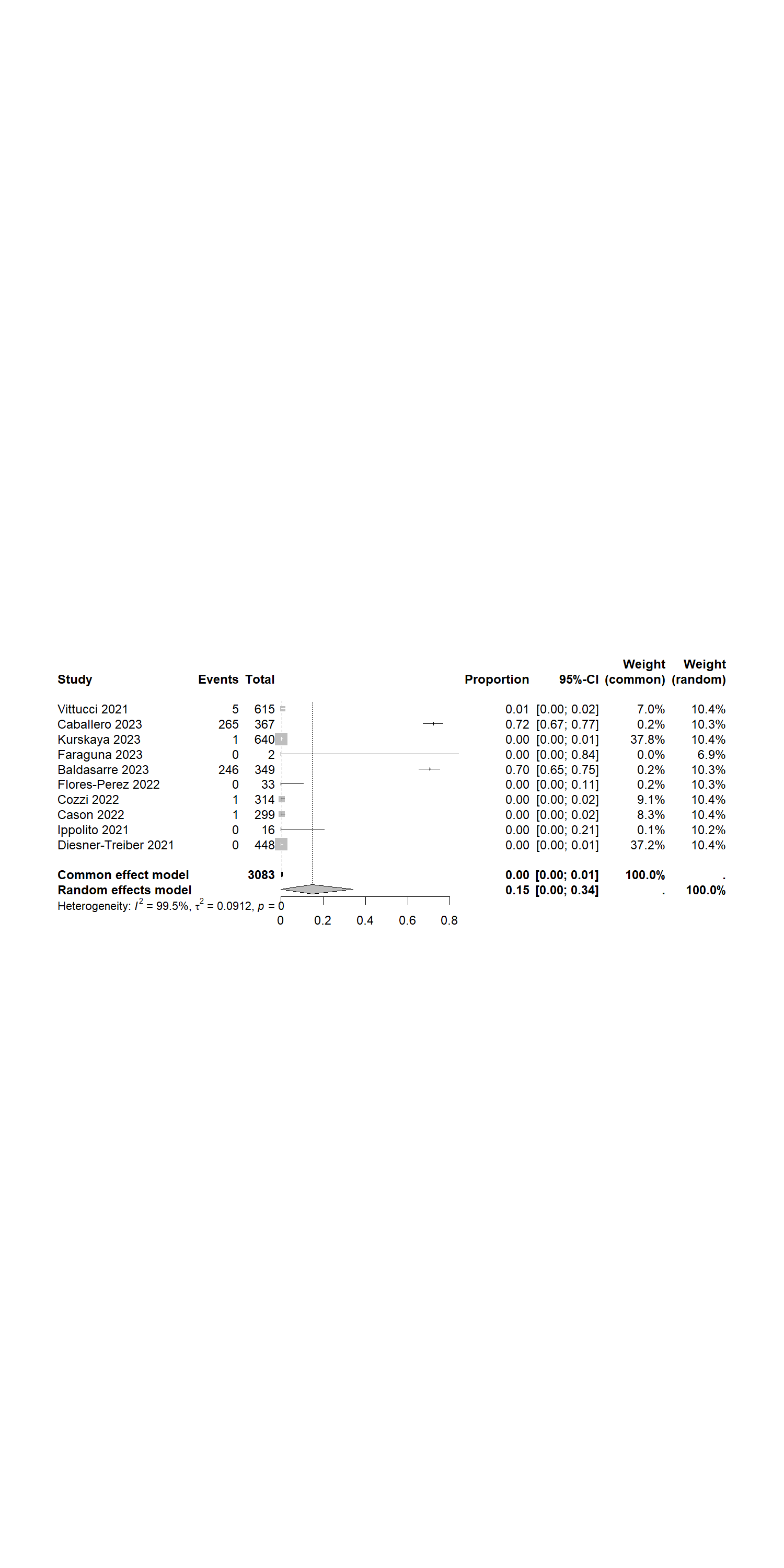


**Figure S11.** Forest Plot of Proportion of Respiratory Syncytial Virus (RSV) during COVID-19


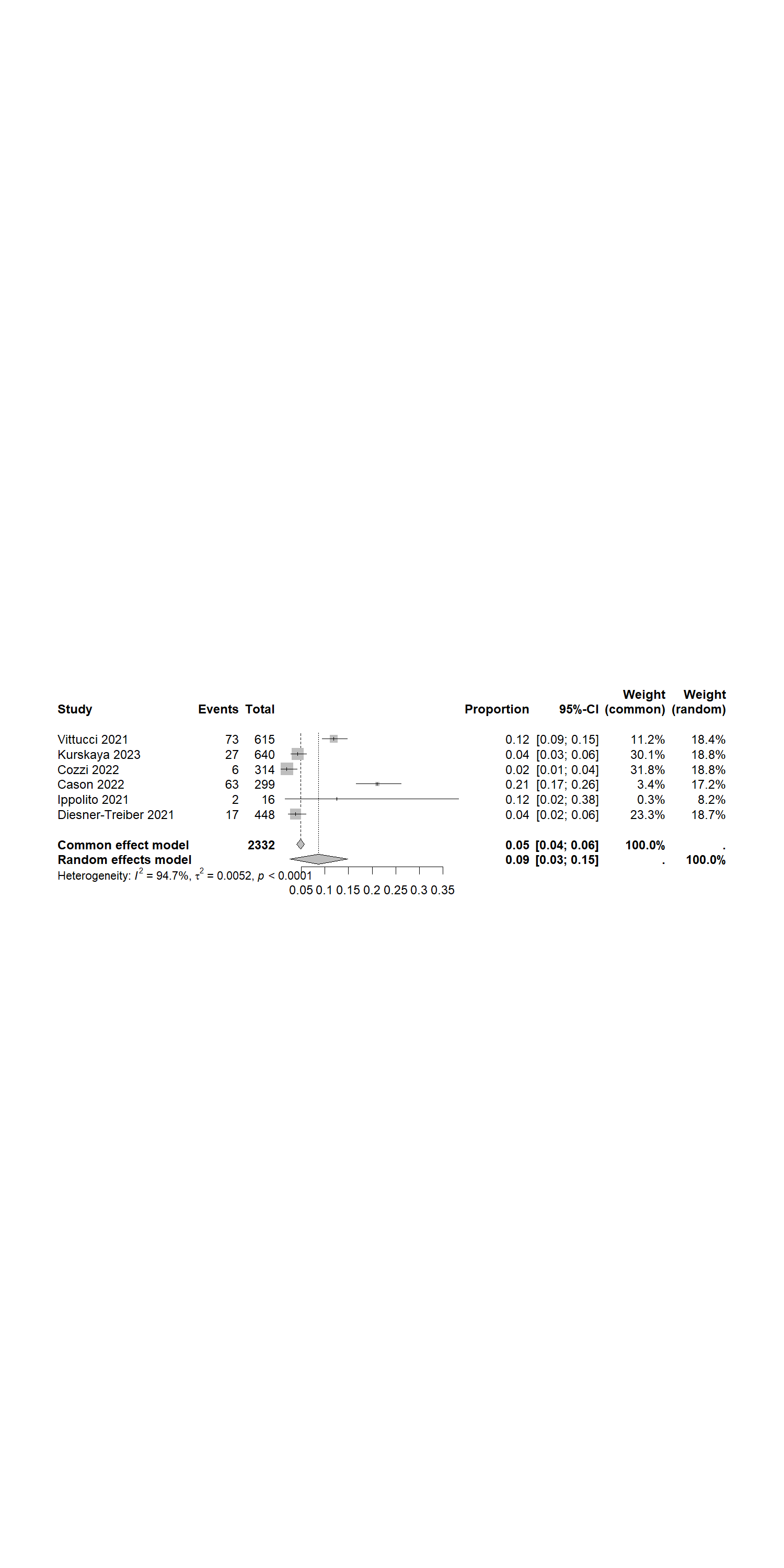


**Figure S12.** Forest Plot of Proportion of Adenovirus (ADV) during COVID-19


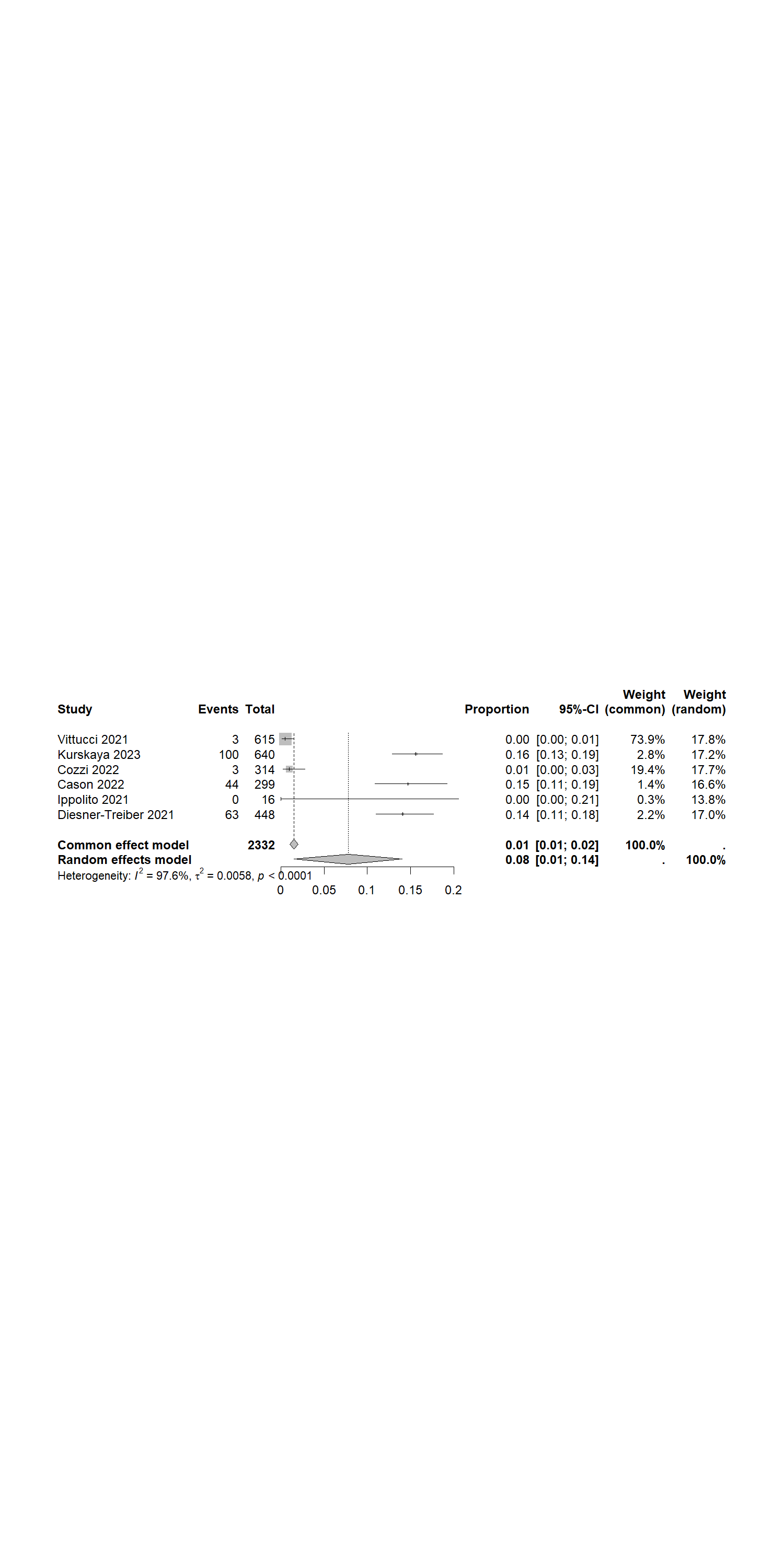


**Figure S13.** Forest Plot of human Coronavirus (hCOV) during COVID-19


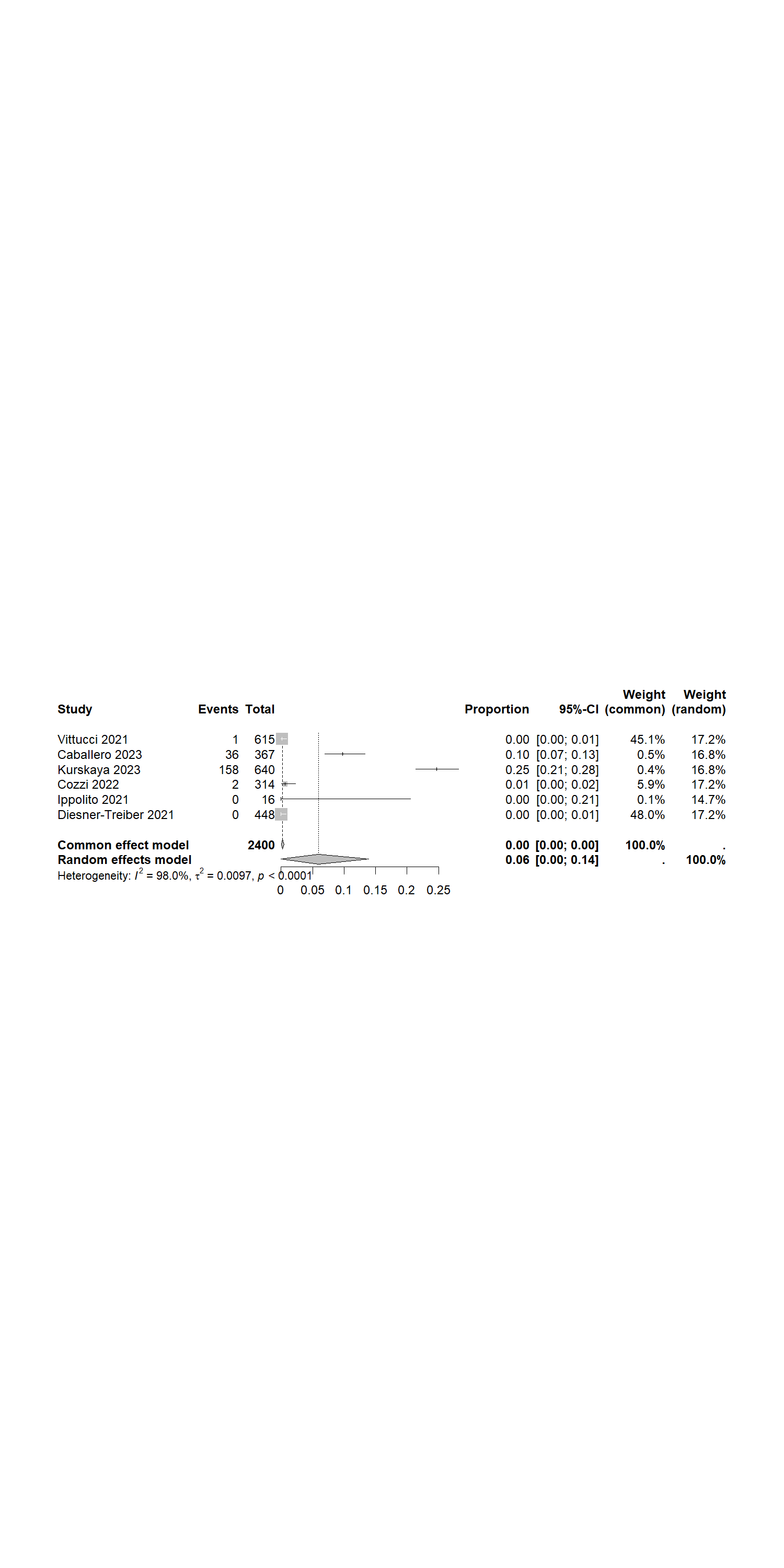


**Figure S14.** Forest Plot of Proportion of Human Metapneumovirus (hMPV) during COVID-19


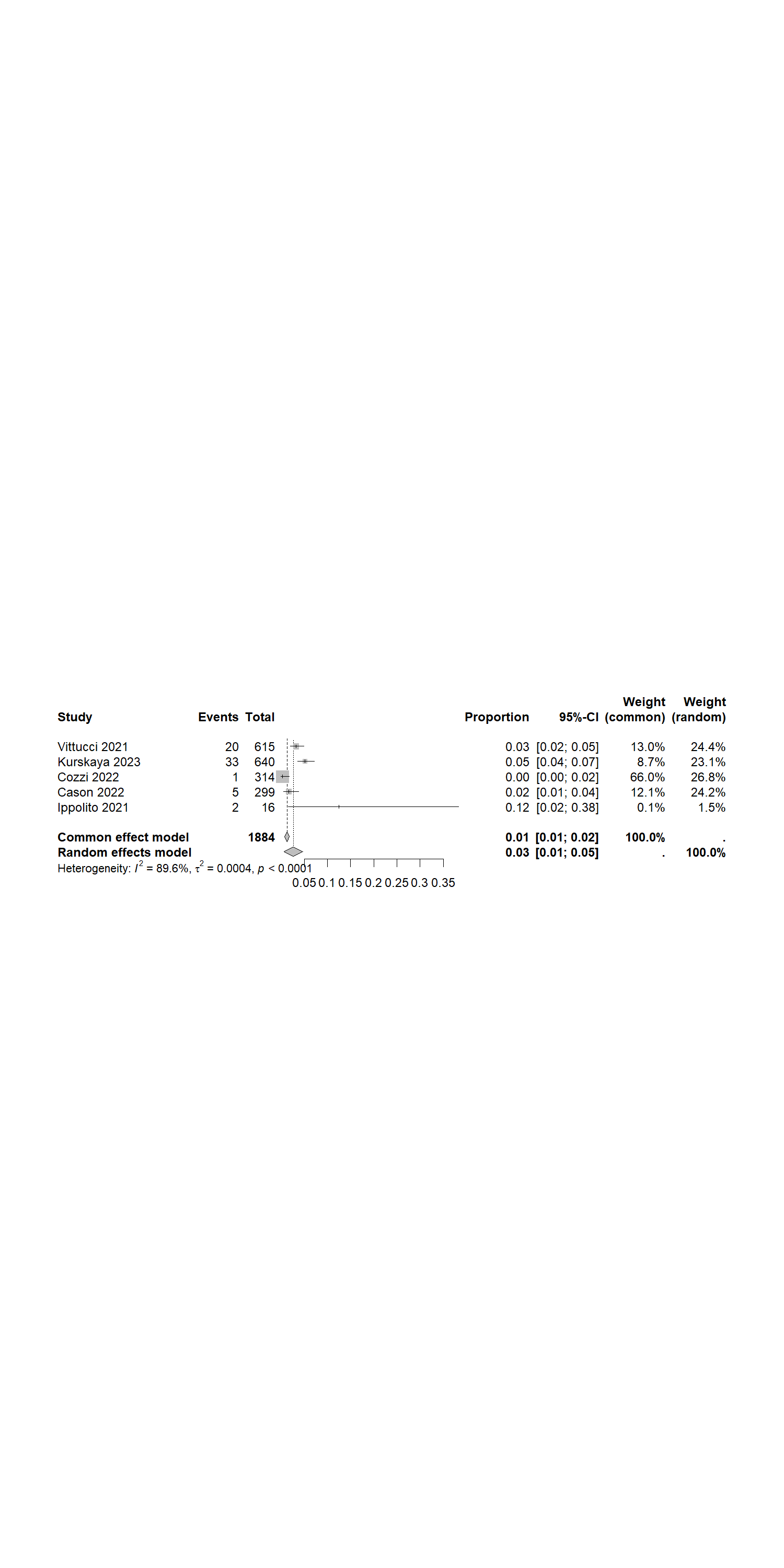


**Figure S15.** Forest Plot of Bocavirus (BoV) during COVID-19


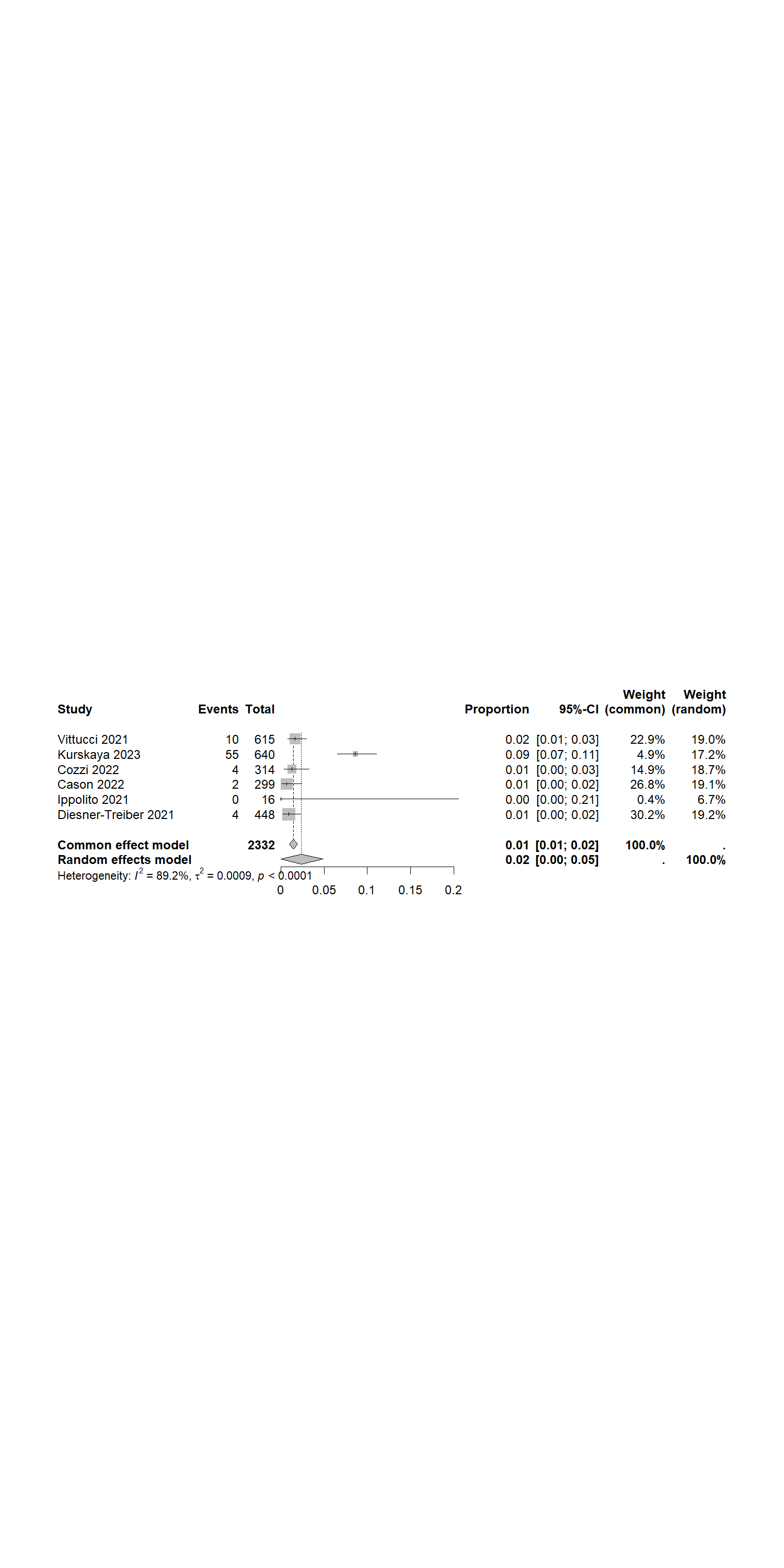


**Figure S16.** Forest Plot of Parainfluenzavirus (PIV) during COVID-19


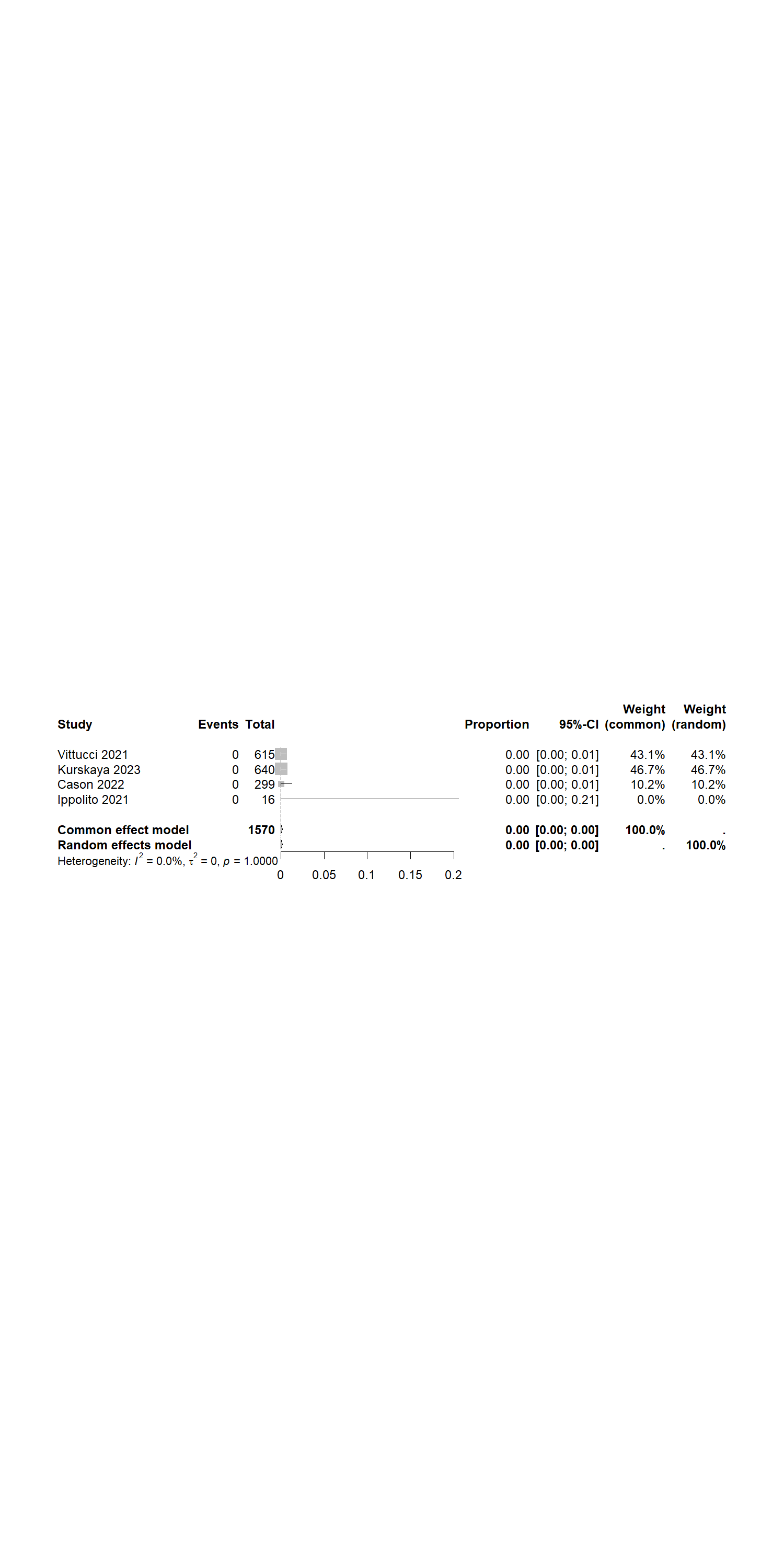


**Figure S17.** Forest Plot of Proportion of Influenza virus (IV) during COVID-19


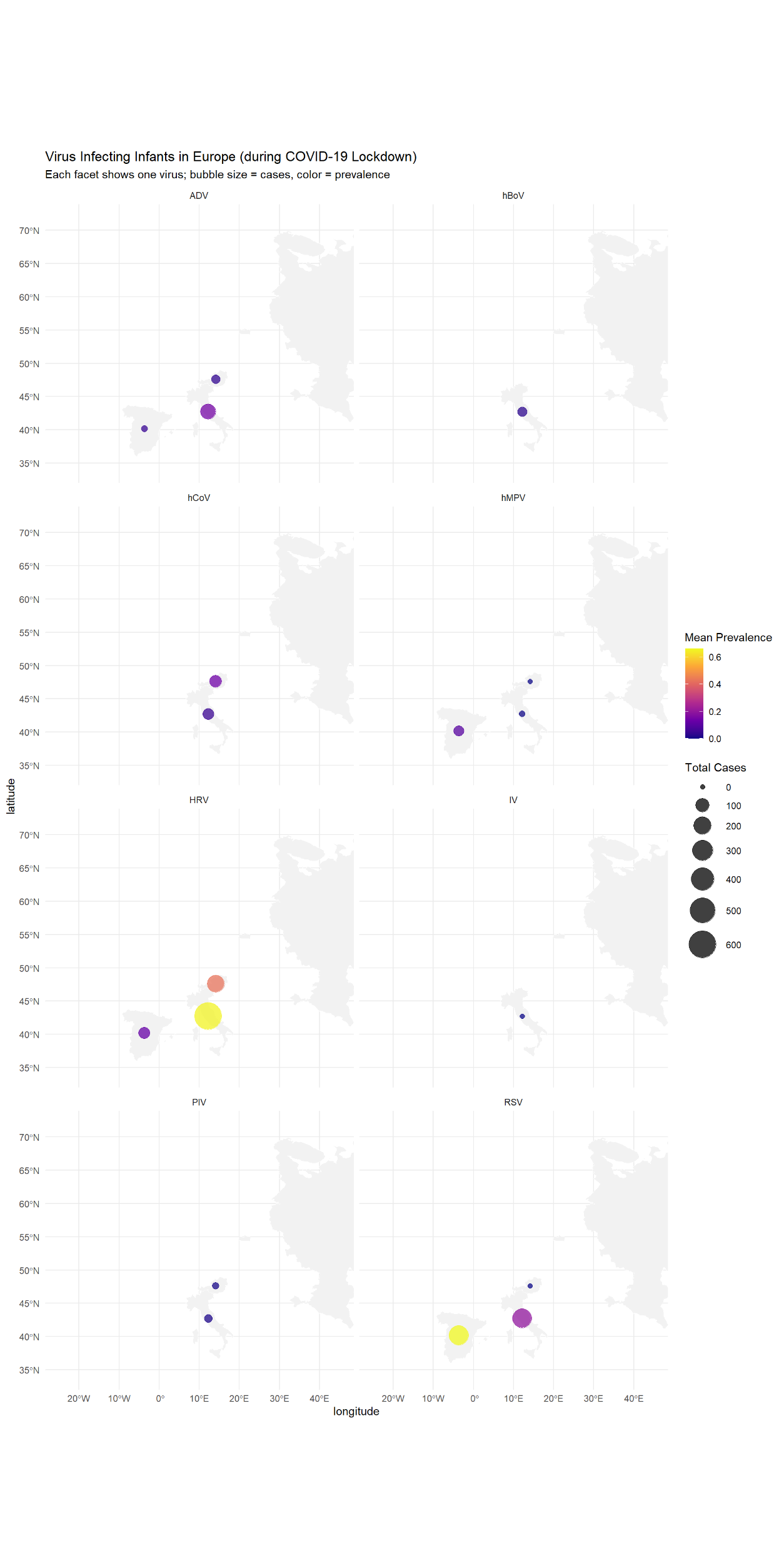


**Figure S18.** Bubble plots for prevalence of viral identifications in infants with respiratory tract infection in European countries during COVID-19 pandemic.


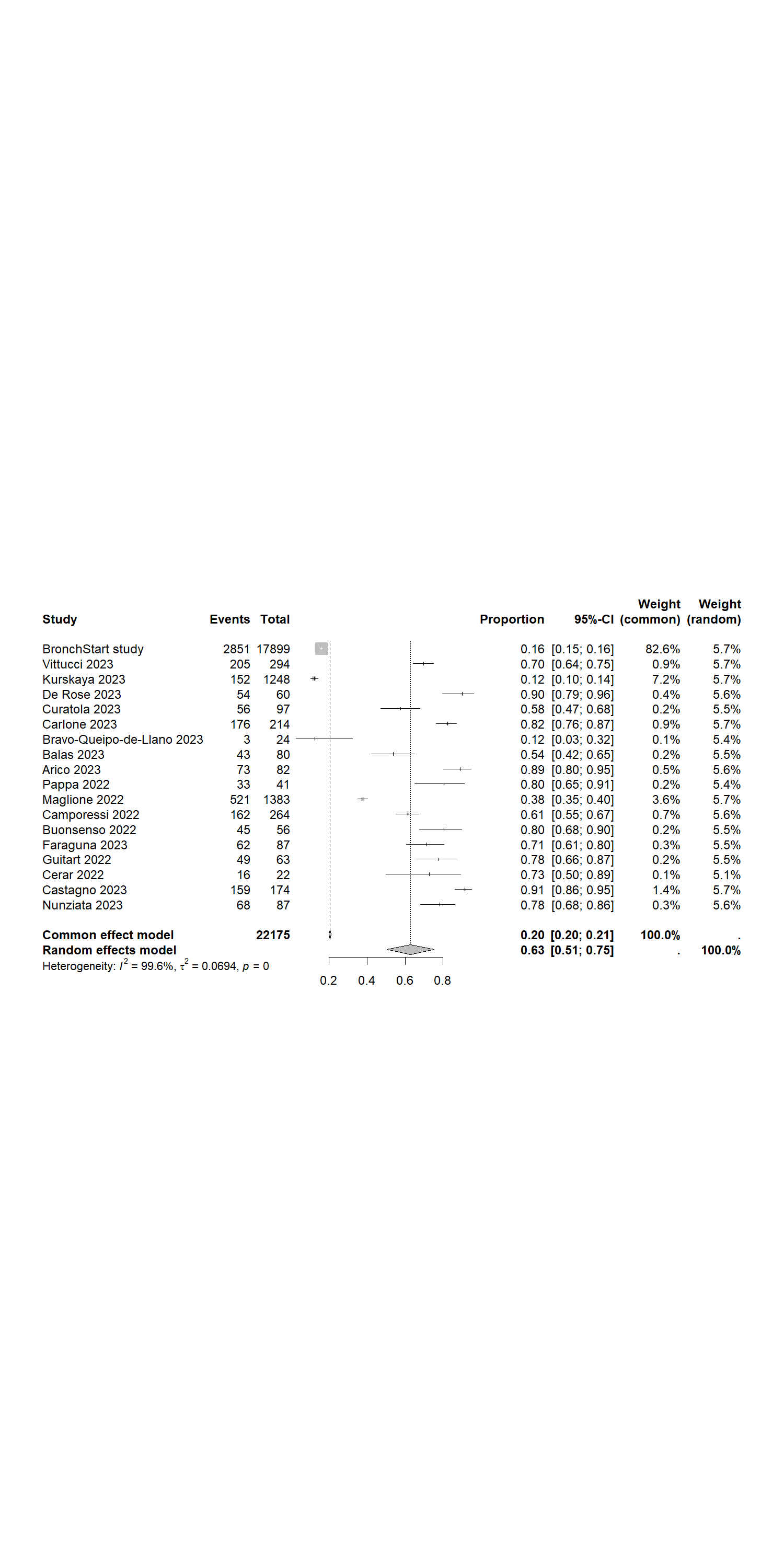


**Figure S19.** Forest Plot of Proportion of Respiratory Syncytial Virus (RSV) after COVID-19


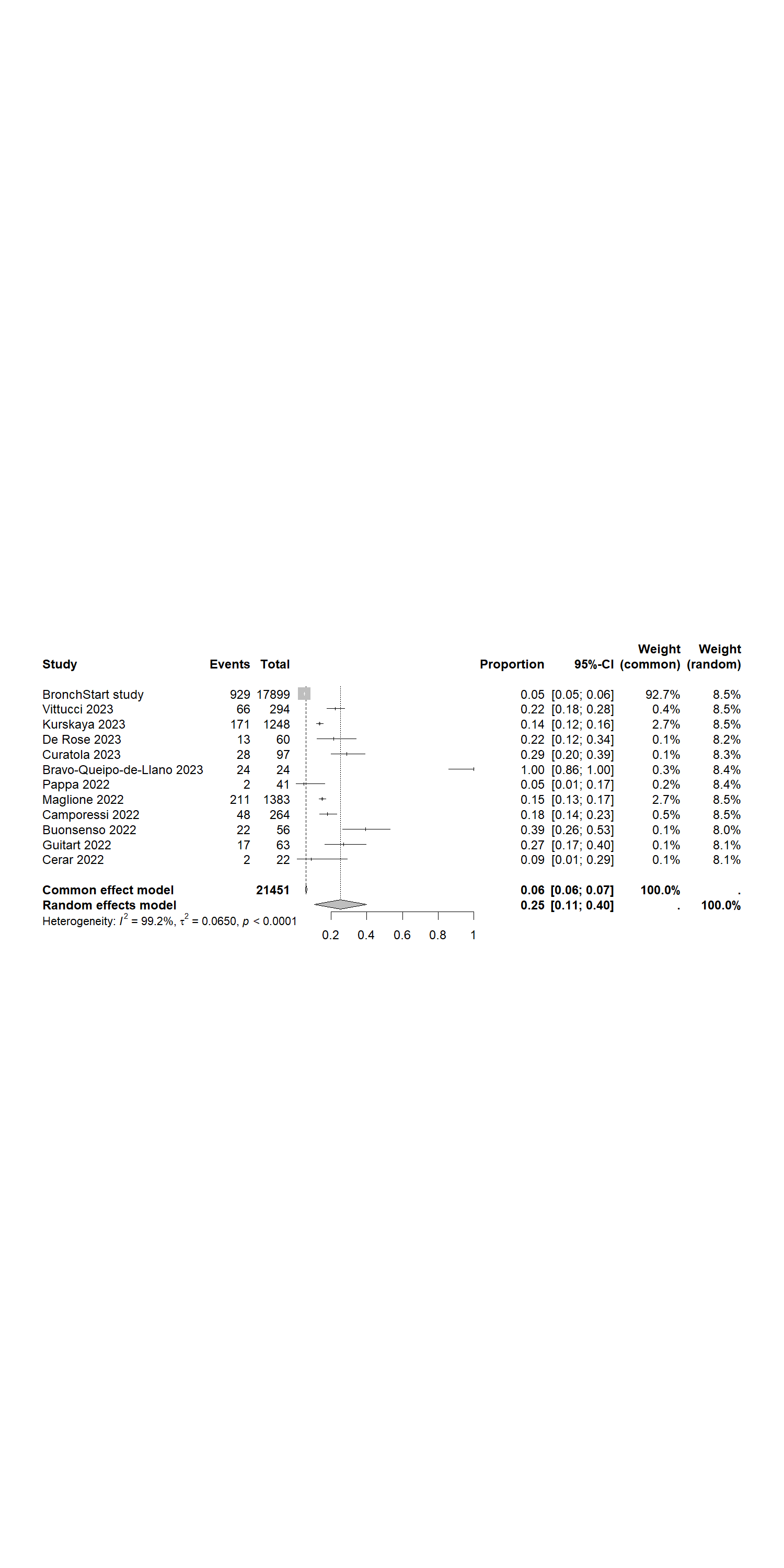


**Figure S20.** Forest Plot of Proportion of Human Rhinovirus (HRV) after COVID-19


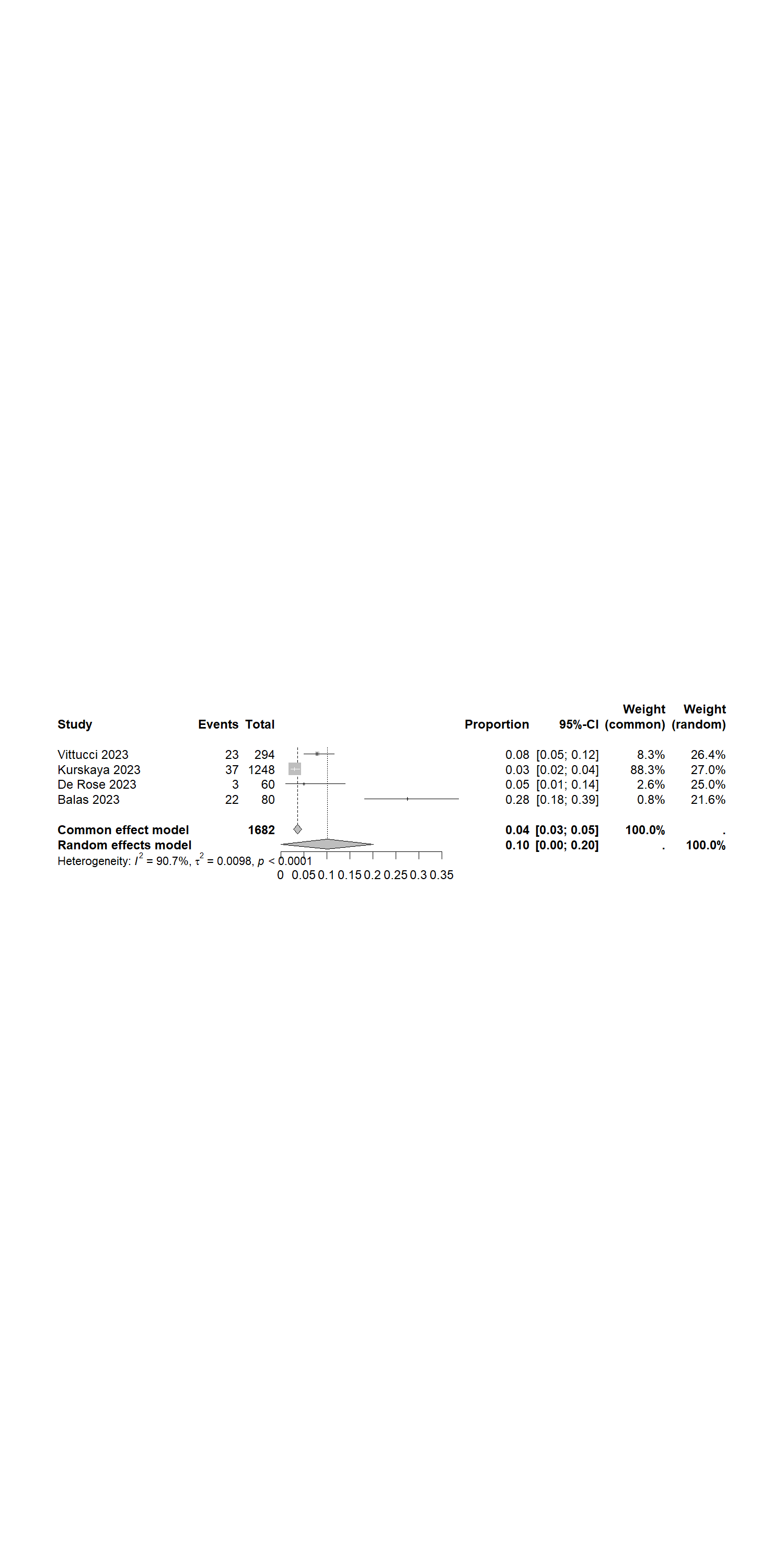


**Figure S21.** Forest Plot of Influenza Virus (IV) after COVID-19


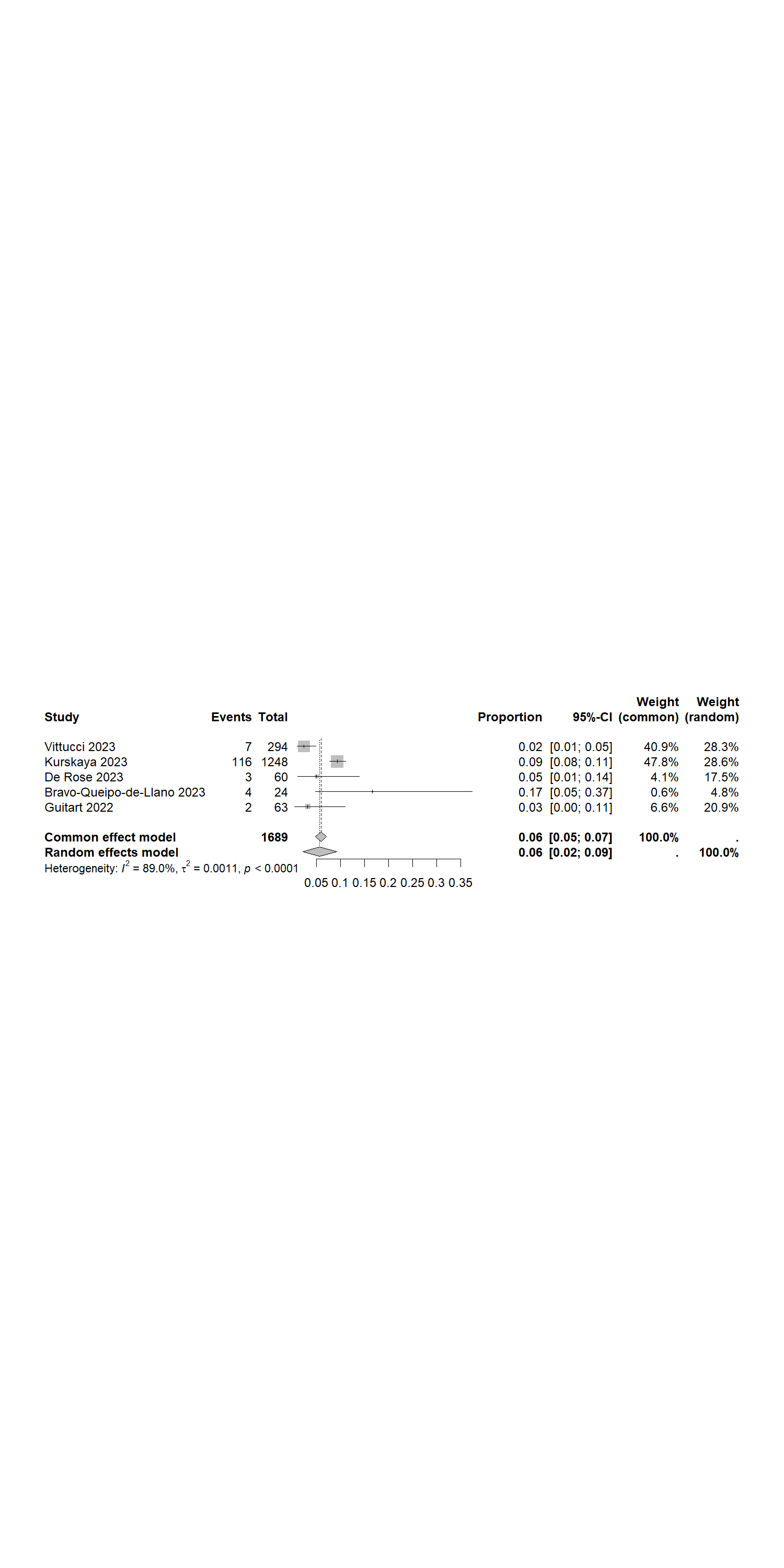


**Figure S22.** Forest Plot of human Coronavirus (hCOV) after COVID-19


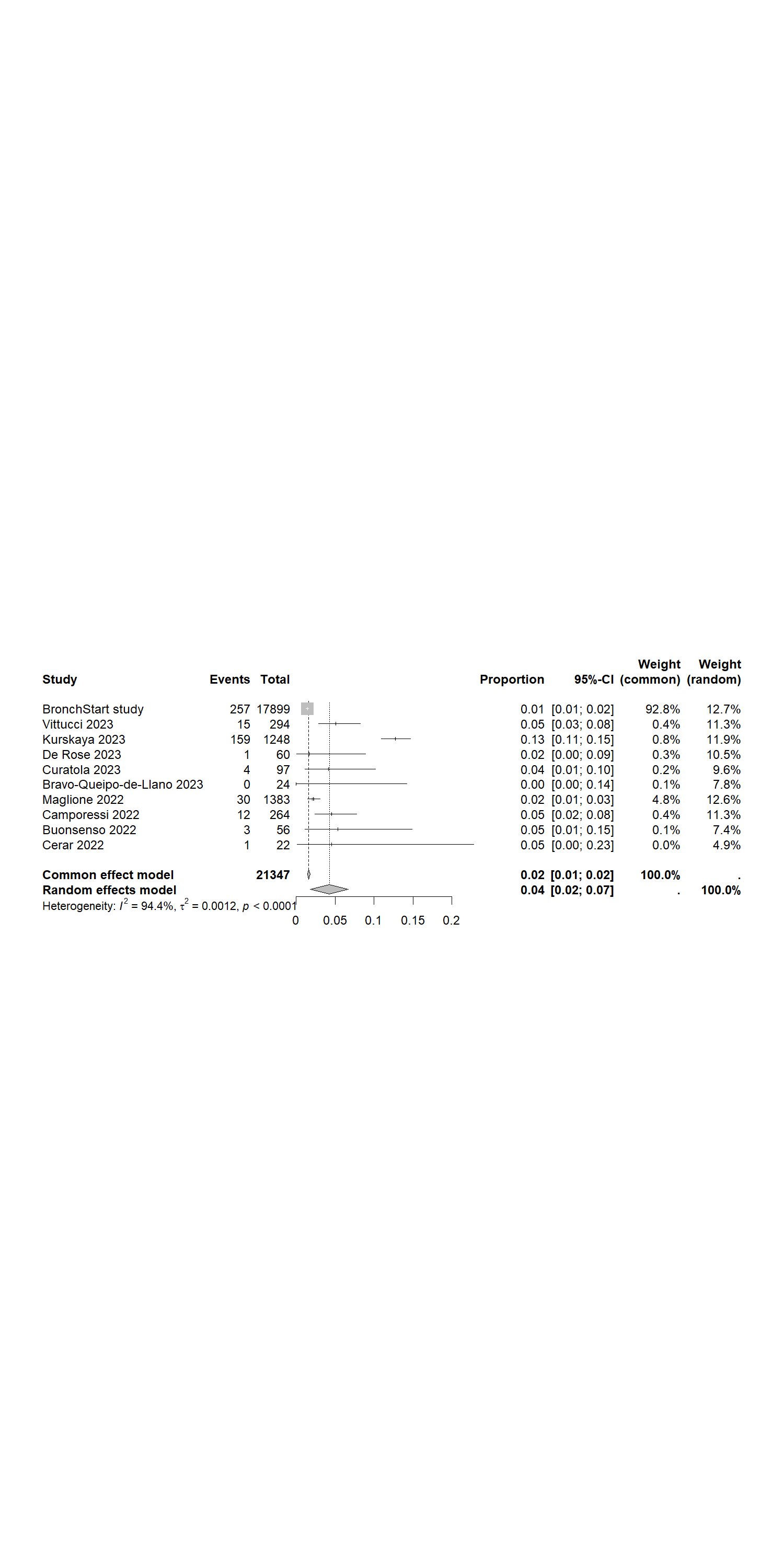


**Figure S23.** Forest Plot of Human Metapneumovirus (hMPV) after COVID-19


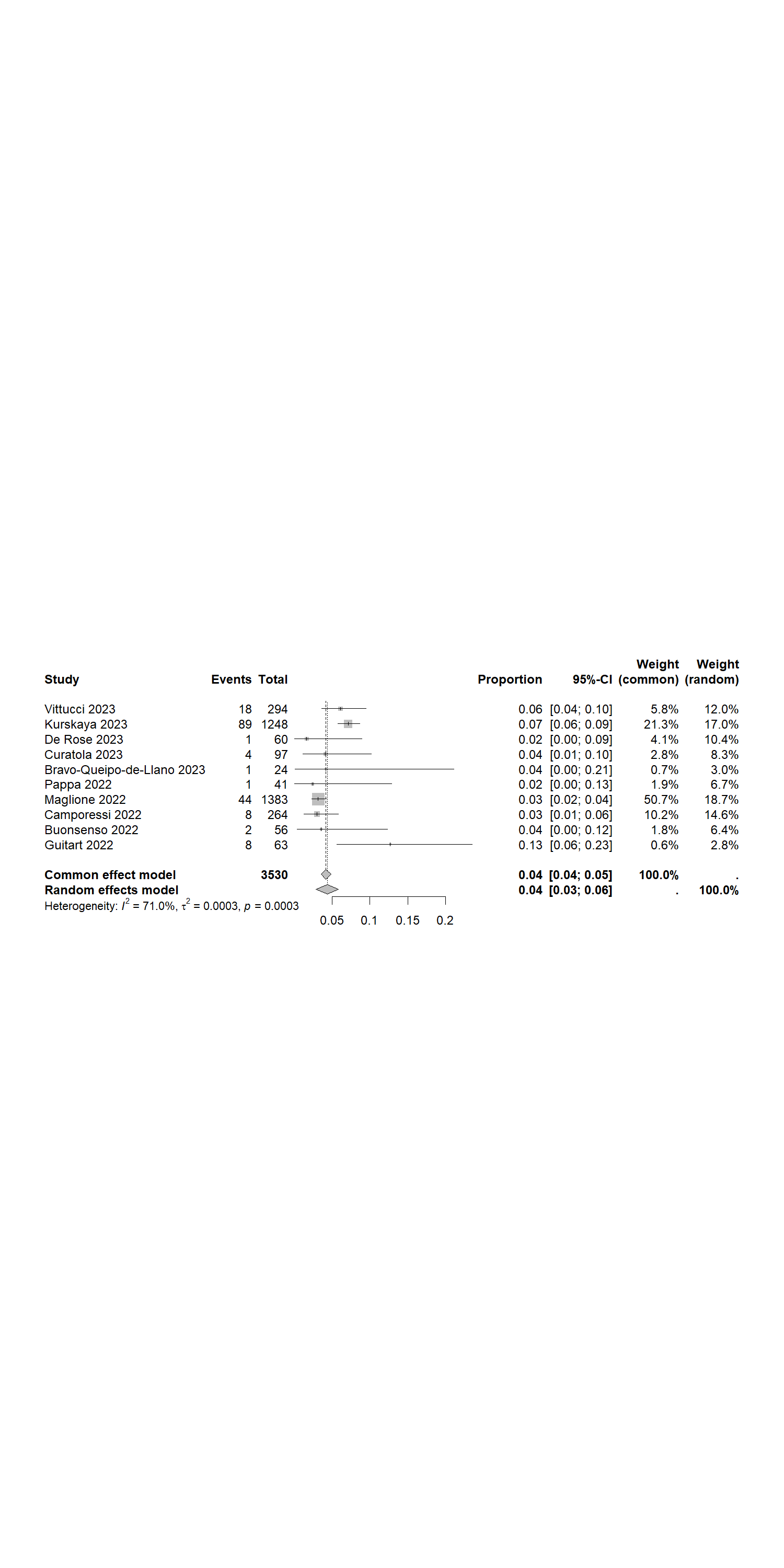


**Figure S24.** Forest Plot of Parainfluenzavirus (PIV) after COVID-19


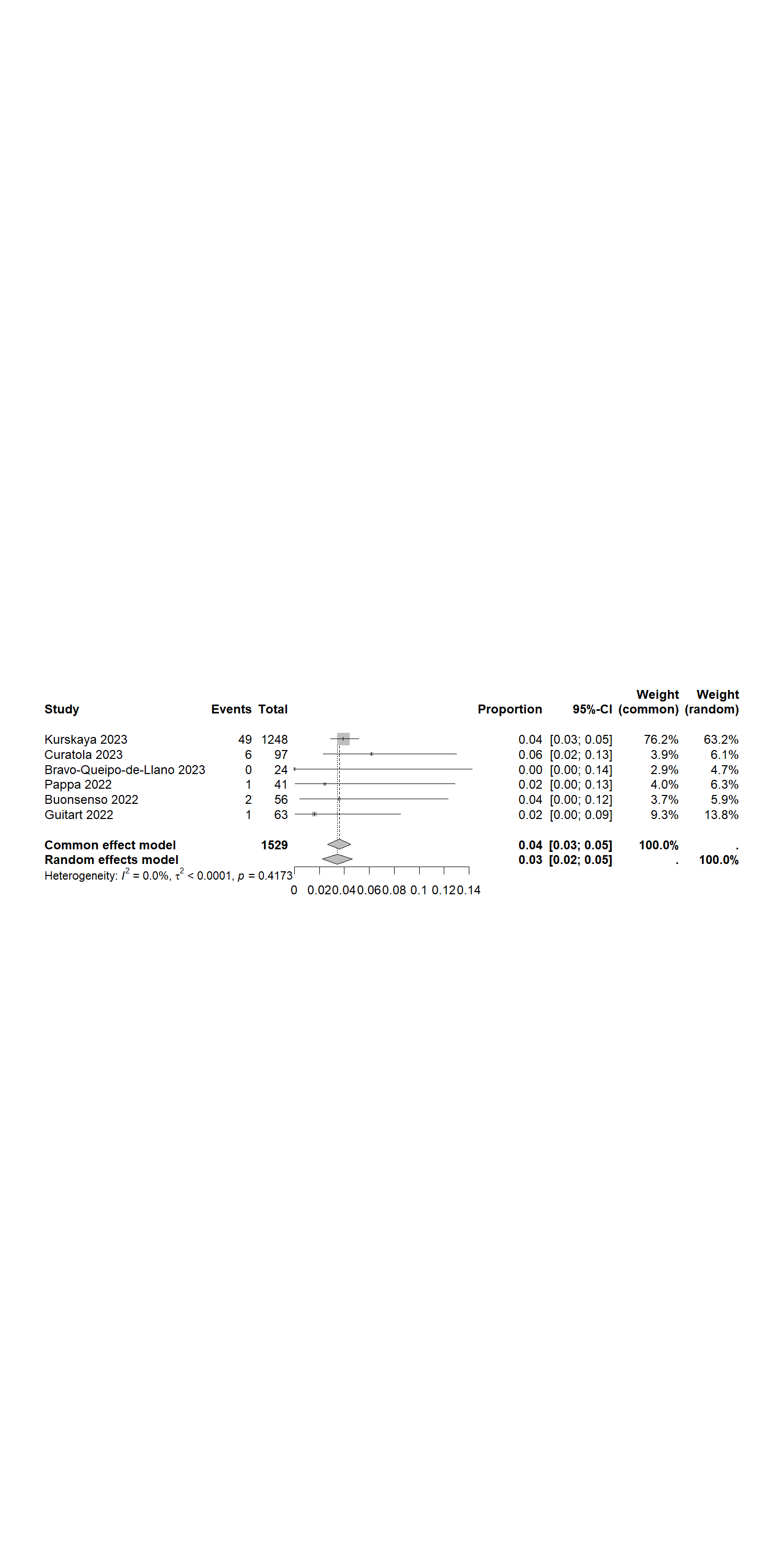


**Figure S25.** Forest Plot of Proportion of Bocavirus (BoV) after COVID-19


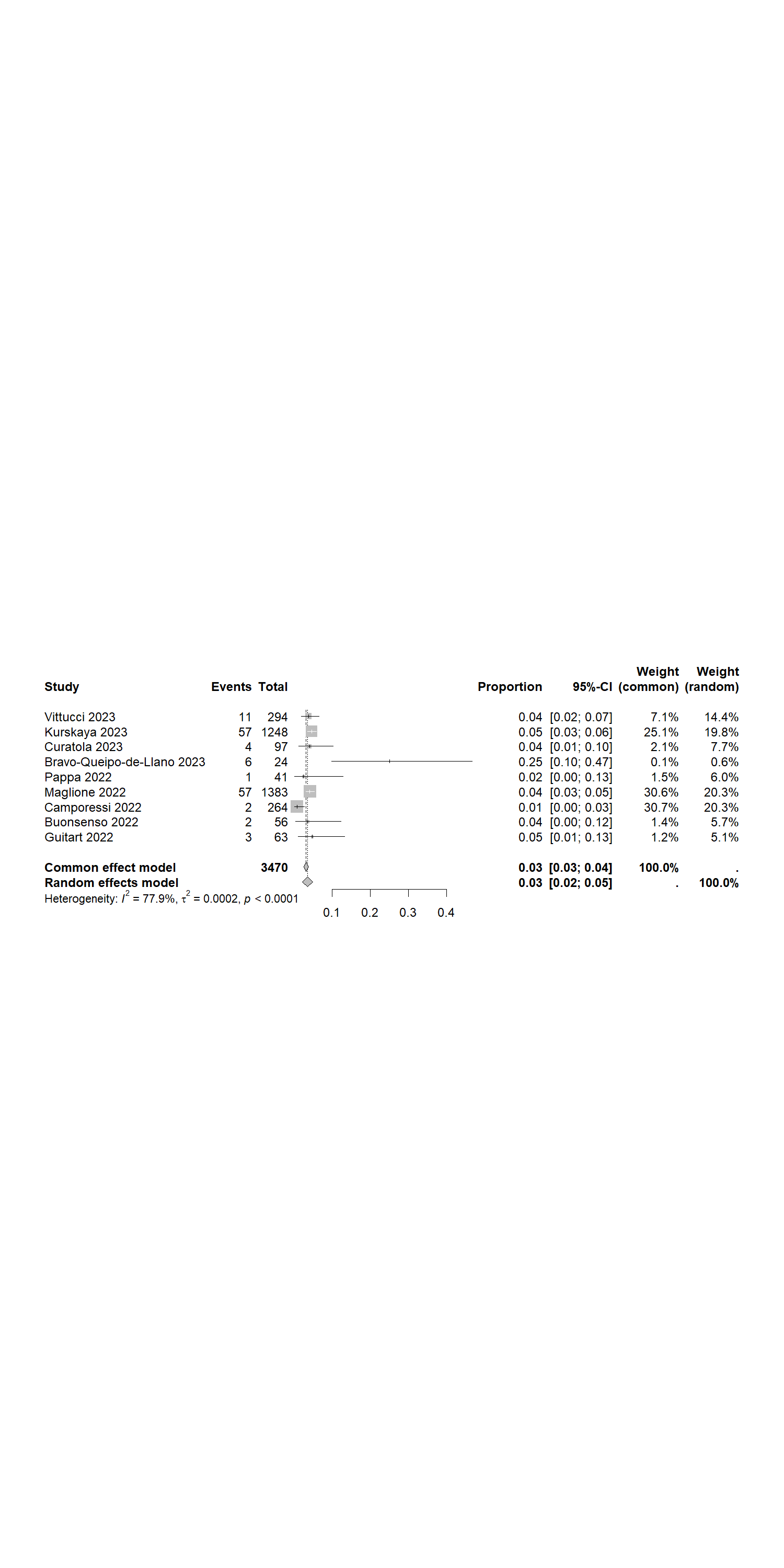


**Figure S26.** Forest Plot of Proportion of Adenovirus (ADV) after COVID-19


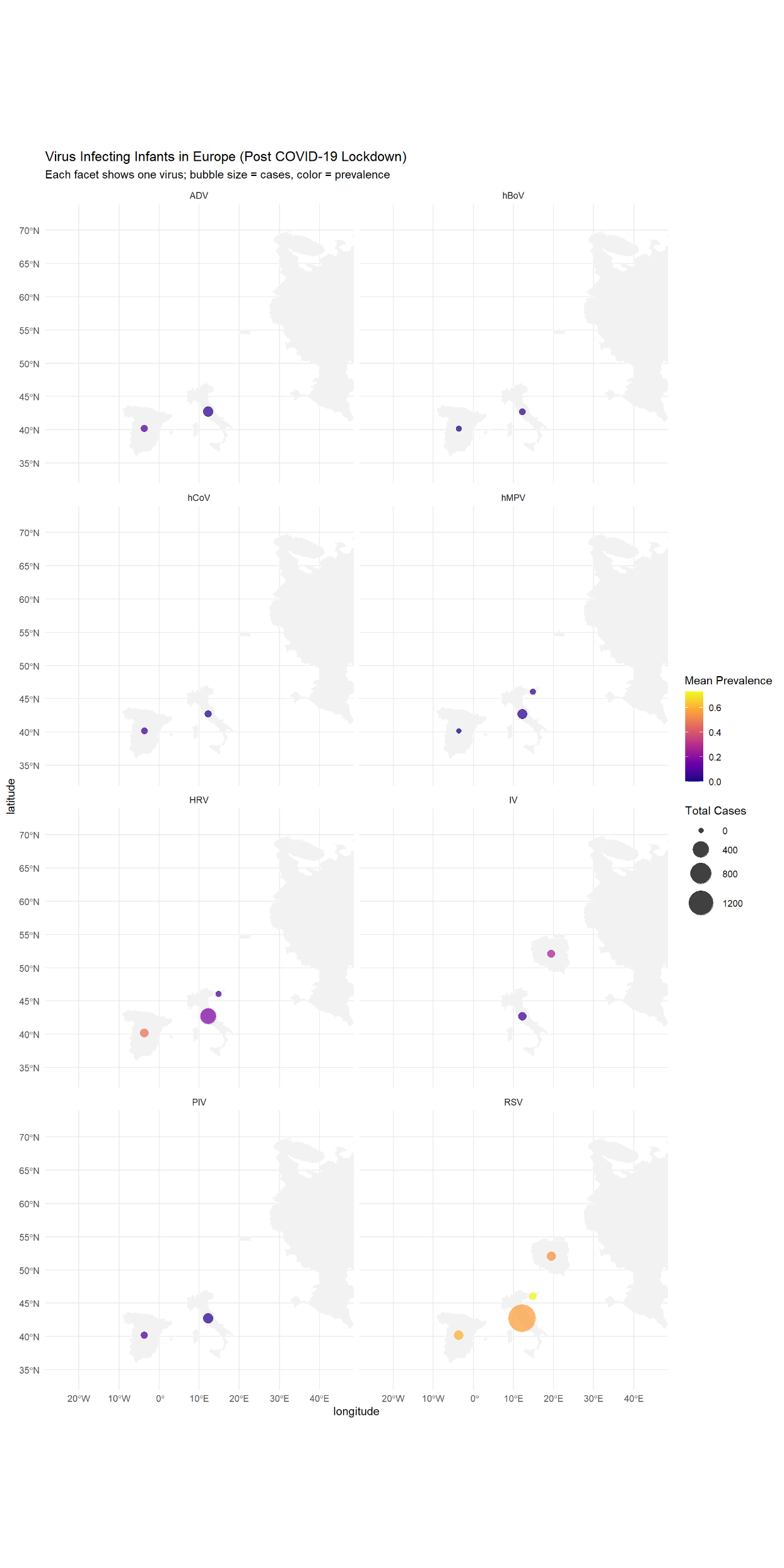


**Figure S27.** Bubble plots for prevalence of viral identifications in infants with respiratory tract infection in European countries after the COVID-19 pandemic.
