# Supplementary material for: Prevalence of common respiratory viruses of infants with respiratory tract infections in European countries in the past decade: a systematic review and meta-analysis comparing between the pre-COVID-19, pandemic, and post-COVID-19 periods
Source: Eur J Pediatr. 2026 May 16;185(6):401. doi: 10.1007/s00431-026-07065-4 (PMC13179882; doi:10.1007/s00431-026-07065-4)
Supplement: Supplementary file 5 — (DOCX 61.2 KB) [file 431_2026_7065_MOESM5_ESM.docx]

**References**

1. Naghavi M, Abajobir AA, Abbafati C, Abbas KM, Abd-Allah F, Abera SF, et al. Global, regional, and national age-sex specific mortality for 264 causes of death, 1980–2016: a systematic analysis for the Global Burden of Disease Study 2016. The Lancet. 2017;390:1151–210. https://doi.org/10.1016/S0140-6736(17)32152-9

2. Bush A. Impact of early life exposures on respiratory disease. Paediatr Respir Rev. 2021;40:24–32. https://doi.org/10.1016/j.prrv.2021.05.006

3. Lopez Bernal JA, Upton MN, Henderson AJ, Dedman D, McCarthy A, Davey Smith G, et al. Lower respiratory tract infection in the first year of life is associated with worse lung function in adult life: prospective results from the Barry Caerphilly Growth study. Ann Epidemiol. 2013;23:422–7. https://doi.org/10.1016/j.annepidem.2013.05.006

4. Carraro S, Scheltema N, Bont L, Baraldi E. Early-life origins of chronic respiratory diseases: understanding and promoting healthy ageing. European Respiratory Journal. 2014;44:1682–96. https://doi.org/10.1183/09031936.00084114

5. Rantala AK, Jaakkola MS, Mäkikyrö EMS, Hugg TT, Jaakkola JJK. Early Respiratory Infections and the Development of Asthma in the First 27 Years of Life. Am J Epidemiol. 2015;182:615–23. https://doi.org/10.1093/aje/kwv093

6. Baraldi E, Bonadies L, Manzoni P. Evidence on the Link between Respiratory Syncytial Virus Infection in Early Life and Chronic Obstructive Lung Diseases. Am J Perinatol. 2020;37:S26–30. https://doi.org/10.1055/s-0040-1714345

7. Matsumoto K, Inoue H. Viral infections in asthma and COPD. Respir Investig. 2014;52:92–100. https://doi.org/10.1016/j.resinv.2013.08.005

8. Jartti T, Gern JE. Role of viral infections in the development and exacerbation of asthma in children. Journal of Allergy and Clinical Immunology. 2017;140:895–906. https://doi.org/10.1016/j.jaci.2017.08.003

9. Bønnelykke K, Vissing NH, Sevelsted A, Johnston SL, Bisgaard H. Association between respiratory infections in early life and later asthma is independent of virus type. Journal of Allergy and Clinical Immunology. 2015;136:81-86.e4. https://doi.org/10.1016/j.jaci.2015.02.024

10. Scotta MC, Chakr VCBG, de Moura A, Becker RG, de Souza APD, Jones MH, et al. Respiratory viral coinfection and disease severity in children: A systematic review and meta-analysis. Journal of Clinical Virology. 2016;80:45–56. https://doi.org/10.1016/j.jcv.2016.04.019

11. Fisman D. Seasonality of viral infections: mechanisms and unknowns. Clinical Microbiology and Infection. 2012;18:946–54. https://doi.org/10.1111/j.1469-0691.2012.03968.x

12. Altizer S, Dobson A, Hosseini P, Hudson P, Pascual M, Rohani P. Seasonality and the dynamics of infectious diseases. Ecol Lett. 2006;9:467–84. https://doi.org/10.1111/j.1461-0248.2005.00879.x

13. Luterbacher J, Dietrich D, Xoplaki E, Grosjean M, Wanner H. European Seasonal and Annual Temperature Variability, Trends, and Extremes Since 1500. Science (1979). 2004;303:1499–503. https://doi.org/10.1126/science.1093877

14. Iftekhar EN, Priesemann V, Balling R, Bauer S, Beutels P, Calero Valdez A, et al. A look into the future of the COVID-19 pandemic in Europe: an expert consultation. The Lancet Regional Health - Europe. 2021;8:100185. https://doi.org/10.1016/j.lanepe.2021.100185

15. Agca H, Akalin H, Saglik I, Hacimustafaoglu M, Celebi S, Ener B. Changing epidemiology of influenza and other respiratory viruses in the first year of COVID-19 pandemic. J Infect Public Health. 2021;14:1186–90. https://doi.org/10.1016/j.jiph.2021.08.004

16. Weinberger Opek M, Yeshayahu Y, Glatman-Freedman A, Kaufman Z, Sorek N, Brosh-Nissimov T. Delayed respiratory syncytial virus epidemic in children after relaxation of COVID-19 physical distancing measures, Ashdod, Israel, 2021. Euro Surveill. NLM (Medline); 2021;26. https://doi.org/10.2807/1560-7917.ES.2021.26.29.2100706

17. Taylor A, Whittaker E. The Changing Epidemiology of Respiratory Viruses in Children During the COVID-19 Pandemic: A Canary in a COVID Time. Pediatric Infectious Disease Journal. 2022;41:e46–8. https://doi.org/10.1097/INF.0000000000003396

18. Shamseer L, Moher D, Clarke M, Ghersi D, Liberati A, Petticrew M, et al. Preferred reporting items for systematic review and meta-analysis protocols (PRISMA-P) 2015: elaboration and explanation. BMJ. 2015;349:g7647–g7647. https://doi.org/10.1136/bmj.g7647

19. Hoy D, Brooks P, Woolf A, Blyth F, March L, Bain C, et al. Assessing risk of bias in prevalence studies: modification of an existing tool and evidence of interrater agreement. J Clin Epidemiol. 2012;65:934–9. https://doi.org/10.1016/j.jclinepi.2011.11.014

20. Higgins JPT, Thompson SG. Quantifying heterogeneity in a meta‐analysis. Stat Med. 2002;21:1539–58. https://doi.org/10.1002/sim.1186

21. Oh H-S, Kim D. SpherWave: An R Package for Analyzing Scattered Spherical Data by Spherical Wavelets. 2007.

22. R Core Team. R: A language and environment for statistical computing. Vienna: R Foundation for Statistical Computing, Vienna, Austria; 2025.

23. Zuurbier RP, Bogaert D, de Steenhuijsen Piters WAA, Arp K, Chu MLJN, Sanders EAM, et al. Asymptomatic Viral Presence in Early Life Precedes Recurrence of Respiratory Tract Infections. Pediatric Infectious Disease Journal. 2023;42:59–65. https://doi.org/10.1097/INF.0000000000003732

24. Wick M, Poshtiban A, Kramer R, Bangert M, Lange M, Wetzke M, et al. Inpatient burden of respiratory syncytial virus in children ≤2 years of age in Germany: A retrospective analysis of nationwide hospitalization data, 2019–2022. Influenza Other Respir Viruses. 2023;17. https://doi.org/10.1111/irv.13211

25. Vittucci AC, Antilici L, Russo C, Musolino AMC, Cristaldi S, Cutrera R, et al. Respiratory syncytial virus: can we still believe that after pandemic bronchiolitis is not a critical issue for public health? Eur J Pediatr. 2023;182:5303–13. https://doi.org/10.1007/s00431-023-05201-y

26. Vasconcelos MK, Meyer Sauteur PM, Keitel K, Santoro R, Egli A, Coslovsky M, et al. Detection of mostly viral pathogens and high proportion of antibiotic treatment initiation in hospitalised children with community-acquired pneumonia in Switzerland – baseline findings from the first two years of the KIDS-STEP trial. Swiss Med Wkly. 2023;153:40040. https://doi.org/10.57187/smw.2023.40040

27. Tabatabai J, Ihling CM, Manuel B, Rehbein RM, Schnee S V, Hoos J, et al. Viral Etiology and Clinical Characteristics of Acute Respiratory Tract Infections in Hospitalized Children in Southern Germany (2014–2018). Open Forum Infect Dis. 2023;10. https://doi.org/10.1093/ofid/ofad110

28. Stobbelaar K, Mangodt TC, Van der Gucht W, Delhaise L, Andries J, Gille V, et al. Risk Factors Associated with Severe RSV Infection in Infants: What Is the Role of Viral Co-Infections? Microbiol Spectr. 2023;11. https://doi.org/10.1128/spectrum.04368-22

29. Piñana M, González-Sánchez A, Andrés C, Abanto M, Vila J, Esperalba J, et al. The emergence, impact, and evolution of human metapneumovirus variants from 2014 to 2021 in Spain. Journal of Infection. 2023;87:103–10. https://doi.org/10.1016/j.jinf.2023.05.004

30. Pierangeli A, Piralla A, Uceda Renteria S, Giacomel G, Lunghi G, Pagani E, et al. Multicenter epidemiological investigation and genetic characterization of respiratory syncytial virus and metapneumovirus infections in the pre-pandemic 2018–2019 season in northern and central Italy. Clin Exp Med. 2022;23:2725–37. https://doi.org/10.1007/s10238-022-00973-3

31. Muñoz-Quiles C, López-Lacort M, Díez-Domingo J, Orrico-Sánchez A. Bronchiolitis, Regardless of Its Etiology and Severity, Is Associated With Increased Risk of Asthma: A Population-Based Study. J Infect Dis. 2023;228:840–50. https://doi.org/10.1093/infdis/jiad093

32. Márquez Caballero J, Cordero Matía ME. Epidemiology of Acute Bronchiolitis in a Third-level Hospital During the COVID-19 Pandemic. Arch Bronconeumol. 2023;59:264–6. https://doi.org/10.1016/j.arbres.2022.11.002

33. Kurskaya OG, Prokopyeva EA, Sobolev IA, Solomatina M V., Saroyan TA, Dubovitskiy NA, et al. Changes in the Etiology of Acute Respiratory Infections among Children in Novosibirsk, Russia, between 2019 and 2022: The Impact of the SARS-CoV-2 Virus. Viruses. 2023;15:934. https://doi.org/10.3390/v15040934

34. Jalving HT, Heimdal I, Valand J, Risnes K, Krokstad S, Nordbø SA, et al. The Burden of Human Bocavirus 1 in Hospitalized Children With Respiratory Tract Infections. J Pediatric Infect Dis Soc. 2023;12:282–9. https://doi.org/10.1093/jpids/piad027

35. Illan Montero J, Berger A, Levy J, Busson L, Hainaut M, Goetghebuer T. Retrospective comparison of respiratory syncytial virus and metapneumovirus clinical presentation in hospitalized children. Pediatr Pulmonol. 2023;58:222–9. https://doi.org/10.1002/ppul.26188

36. Hernández-Villarroel AC, Ruiz-García A, Manzanaro C, Echevarría-Zubero R, Bote-Gascón P, Gonzalez-Bertolin I, et al. Lung Ultrasound: A Useful Prognostic Tool in the Management of Bronchiolitis in the Emergency Department. J Pers Med. 2023;13:1624. https://doi.org/10.3390/jpm13121624

37. Hartiala M, Lahti E, Toivonen L, Waris M, Ruuskanen O, Peltola V. Biomarkers of viral and bacterial infection in rhinovirus pneumonia. Front Pediatr. 2023;11. https://doi.org/10.3389/fped.2023.1137777

38. Fonseca MJ, Hagenaars S, Bangert M, Flach C, Hudson RDA. Respiratory Syncytial Virus Hospital Admission Rates and Patients’ Characteristics Before the Age of 2 Years in England, 2015–2019. Pediatric Infectious Disease Journal. 2024; https://doi.org/10.1097/INF.0000000000004467

39. Fischer N, Moreels S, Dauby N, Reynders M, Petit E, Gérard M, et al. Influenza versus other respiratory viruses – assessing severity among hospitalised children, Belgium, 2011 to 2020. Eurosurveillance. 2023;28. https://doi.org/10.2807/1560-7917.ES.2023.28.29.2300056

40. Dervaux B, Van Berleere M, Lenne X, Wyckaert M, Dubos F. Impact of RSV test positivity, patient characteristics, and treatment characteristics on the cost of hospitalization for acute bronchiolitis in a French university medical center (2010–2015). Front Pediatr. 2023;11. https://doi.org/10.3389/fped.2023.1126229

41. De Maio F, Fiori B, Bianco DM, Sanguinetti M, Sali M. Respiratory viruses in the pre and post‐pandemic periods in an Italian tertiary hospital. Immun Inflamm Dis. 2023;11. https://doi.org/10.1002/iid3.909

42. De Luca M, D’Amore C, Romani L, Tripiciano C, Clemente V, Mercadante S, et al. Severe viral respiratory infections in the pre‐COVID era: A 5‐year experience in two pediatric intensive care units in Italy. Influenza Other Respir Viruses. 2023;17. https://doi.org/10.1111/irv.13038

43. Ciofi degli Atti M, Rizzo C, D’Amore C, Ravà L, Reale A, Barbieri MA, et al. Acute respiratory infection emergency access in a tertiary care children hospital in Italy, prior and after the SARS‐CoV‐2 emergence. Influenza Other Respir Viruses. 2023;17. https://doi.org/10.1111/irv.13102

44. Bermúdez-Barrezueta L, López-Casillas P, Rojo-Rello S, Sáez-García L, Marugán-Miguelsanz JM, Pino-Vázquez M de la A. Outcomes of viral coinfections in infants hospitalized for acute bronchiolitis. Virol J. 2023;20:235. https://doi.org/10.1186/s12985-023-02197-7

45. Barbieri E, Cavagnis S, Scamarcia A, Cantarutti L, Bertizzolo L, Bangert M, et al. Assessing the burden of bronchiolitis and lower respiratory tract infections in children ≤24 months of age in Italy, 2012–2019. Front Pediatr. 2023;11. https://doi.org/10.3389/fped.2023.1143735

46. Abbate F, Depietri G, Tinelli C, Massimetti G, Picariello S, Peroni D, et al. Impact of the publication of the Italian guidelines for bronchiolitis on the management of hospitalized children in Pisa, Italy. Pediatr Pulmonol. 2023;58:2267–74. https://doi.org/10.1002/ppul.26460

47. Wrotek A, Robakiewicz J, Pawlik K, Rudzinski P, Pilarska I, Jaroń A, et al. The Etiology of Community-Acquired Pneumonia Correlates with Serum Inflammatory Markers in Children. J Clin Med. 2022;11:5506. https://doi.org/10.3390/jcm11195506

48. Vihikangas T, Palmu S, Koivisto A-M, Heikkilä P. Changes in Bronchiolitis Incidence During the Last Two Decades in Tampere, Finland: A Retrospective Study. Pediatric Infectious Disease Journal. 2022;41:867–71. https://doi.org/10.1097/INF.0000000000003662

49. Tabatabai J, Ihling CM, Rehbein RM, Schnee SV, Hoos J, Pfeil J, et al. Molecular epidemiology of respiratory syncytial virus in hospitalised children in Heidelberg, Southern Germany, 2014–2017. Infection, Genetics and Evolution. 2022;98:105209. https://doi.org/10.1016/j.meegid.2022.105209

50. Rybak A, Ouldali N, Angoulvant F, Minodier P, Biscardi S, Madhi F, et al. Shift in Clinical Profile of Hospitalized Pneumonia in Children in the Non-pharmaceutical Interventions Period During the COVID-19 Pandemic: A Prospective Multicenter Study. Front Pediatr. 2022;10. https://doi.org/10.3389/fped.2022.782894

51. Powell E, Sumner E, Shaw AG, Calvez R, Fink CG, Kroll JS. The temporal pattern and lifestyle associations of respiratory virus infection in a cohort study spanning the first two years of life. BMC Pediatr. 2022;22:166. https://doi.org/10.1186/s12887-022-03215-3

52. Nardi S, Carolis L, Iannini R, De Sandro MV, Solito G, Calafatti M, et al. Usefulness of rapid molecular tests in pediatric respiratory tract infections. Ital J Pediatr. 2022;48:21. https://doi.org/10.1186/s13052-022-01200-1

53. Mrcela D, Markic J, Zhao C, Viskovic DV, Milic P, Copac R, et al. Changes following the Onset of the COVID-19 Pandemic in the Burden of Hospitalization for Respiratory Syncytial Virus Acute Lower Respiratory Infection in Children under Two Years: A Retrospective Study from Croatia. Viruses. 2022;14:2746. https://doi.org/10.3390/v14122746

54. Milani GP, Cafora M, Favero C, Luganini A, Carugno M, Lenzi E, et al. PM2 .5, PM10 and bronchiolitis severity: A cohort study. Pediatric Allergy and Immunology. 2022;33. https://doi.org/10.1111/pai.13853

55. McGeoch LJ, Thornton H V., Blair PS, Christensen H, Turner NL, Muir P, et al. Prognostic value of upper respiratory tract microbes in children presenting to primary care with respiratory infections: A prospective cohort study. PLoS One. 2022;17:e0268131. https://doi.org/10.1371/journal.pone.0268131

56. Matera L, Nenna R, Frassanito A, Petrarca L, Mancino E, Rizzo V, et al. Low lymphocyte count: A clinical severity marker in infants with bronchiolitis. Pediatr Pulmonol. 2022;57:1770–5. https://doi.org/10.1002/ppul.25919

57. Lumley SF, Richens N, Lees E, Cregan J, Kalimeris E, Oakley S, et al. Changes in paediatric respiratory infections at a UK teaching hospital 2016–2021; impact of the SARS-CoV-2 pandemic. Journal of Infection. 2022;84:40–7. https://doi.org/10.1016/j.jinf.2021.10.022

58. Langley JM, Bianco V, Domachowske JB, Madhi SA, Stoszek SK, Zaman K, et al. Incidence of Respiratory Syncytial Virus Lower Respiratory Tract Infections During the First 2 Years of Life: A Prospective Study Across Diverse Global Settings. J Infect Dis. 2022;226:374–85. https://doi.org/10.1093/infdis/jiac227

59. Johannesen CK, van Wijhe M, Tong S, Fernández L V, Heikkinen T, van Boven M, et al. Age-Specific Estimates of Respiratory Syncytial Virus-Associated Hospitalizations in 6 European Countries: A Time Series Analysis. J Infect Dis. 2022;226:S29–37. https://doi.org/10.1093/infdis/jiac150

60. Hussain F, Delgado Thompson M, Vick D, West J, Edwards M. Clinical severity of RSV bronchiolitis. Health Sci Rep. 2022;5. https://doi.org/10.1002/hsr2.543

61. Heppe Montero M, Gil-Prieto R, Walter S, Aleixandre Blanquer F, Gil De Miguel Á. Burden of severe bronchiolitis in children up to 2 years of age in Spain from 2012 to 2017. Hum Vaccin Immunother. 2022;18. https://doi.org/10.1080/21645515.2021.1883379

62. García‐Arroyo L, Prim N, Del Cuerpo M, Marín P, Roig MC, Esteban M, et al. Prevalence and seasonality of viral respiratory infections in a temperate climate region: A 24‐year study (1997–2020). Influenza Other Respir Viruses. 2022;16:756–66. https://doi.org/10.1111/irv.12972

63. Cerar S, Pirnovar V. A Comparative Analysis of the Occurrence of Lower Respiratory Tract Infections Caused by Respiratory Syncytial Virus among Newborns in the Years before and during Covid-19 Pandemic at a Tertiary Referral Hospital in Slovenia. Central European Journal of Paediatrics. University Clinical Hospital Tuzla; 2022;18:100–7. https://doi.org/10.5457/p2005-114.322

64. Cerar S, Kučan R, Paro-Panjan D, Nosan G. The burden of viral lower respiratory tract infections during the neonatal period: six-year experience at a tertiary referral hospital. Croat Med J. 2022;63:343–51. https://doi.org/10.3325/cmj.2022.63.343

65. Caini S, Stolyarov K, Sominina A, Smorodintseva E, Staadegaard L, Paget J, et al. A comparative analysis of the epidemiology of influenza and respiratory syncytial virus in Russia, 2013/14 to 2018/19. J Glob Health. 2022;12:04009. https://doi.org/10.7189/jogh.12.04009

66. Kohns Vasconcelos M, Loens K, Sigfrid L, Iosifidis E, Epalza C, Donà D, et al. Aetiology of acute respiratory infection in preschool children requiring hospitalisation in Europe—results from the PED-MERMAIDS multicentre case–control study. BMJ Open Respir Res. 2021;8:e000887. https://doi.org/10.1136/bmjresp-2021-000887

67. Torres-Fernandez D, Casellas A, Mellado MJ, Calvo C, Bassat Q. Acute bronchiolitis and respiratory syncytial virus seasonal transmission during the COVID-19 pandemic in Spain: A national perspective from the pediatric Spanish Society (AEP). Journal of Clinical Virology. 2021;145:105027. https://doi.org/10.1016/j.jcv.2021.105027

68. Sominina A, Danilenko D, Komissarov A, Pisareva M, Musaeva T, Bakaev M, et al. Age-Specific Etiology of Severe Acute Respiratory Infections and Influenza Vaccine Effectivity in Prevention of Hospitalization in Russia, 2018–2019 Season. J Epidemiol Glob Health. 2021;11:413–25. https://doi.org/10.1007/s44197-021-00009-1

69. Petat H, Gajdos V, Angoulvant F, Vidalain P-O, Corbet S, Marguet C, et al. High Frequency of Viral Co-Detections in Acute Bronchiolitis. Viruses. 2021;13:990. https://doi.org/10.3390/v13060990

70. Penela-Sánchez D, González-de-Audicana J, Armero G, Henares D, Esteva C, de-Sevilla M-F, et al. Lower Respiratory Tract Infection and Genus Enterovirus in Children Requiring Intensive Care: Clinical Manifestations and Impact of Viral Co-Infections. Viruses. 2021;13:2059. https://doi.org/10.3390/v13102059

71. Oh D-Y, Biere B, Grenz M, Wolff T, Schweiger B, Dürrwald R, et al. Virological Surveillance and Molecular Characterization of Human Parainfluenzavirus Infection in Children with Acute Respiratory Illness: Germany, 2015–2019. Microorganisms. 2021;9:1508. https://doi.org/10.3390/microorganisms9071508

72. Sans Munoz I, Bachiller Luque MR, Eiros Bouza JM. Infecciones víricas del tracto respiratorio. Pediatr Integral [Internet]. 2021;25:13–20. https://syndromictrends.com/metric/panel/rp/percent_positivity/

73. Mattila J, Thomas E, Lehtinen P, Vuorinen T, Waris M, Heikkinen T. Burden of influenza during the first year of life. Influenza Other Respir Viruses. 2021;15:506–12. https://doi.org/10.1111/irv.12820

74. Ljubin-Sternak S, Slović A, Mijač M, Jurković M, Forčić D, Ivković-Jureković I, et al. Prevalence and Molecular Characterization of Human Bocavirus Detected in Croatian Children with Respiratory Infection. Viruses. 2021;13:1728. https://doi.org/10.3390/v13091728

75. Korsun N, Angelova S, Trifonova I, Voleva S, Grigorova I, Tzotcheva I, et al. Predominance of ON1 and BA9 genotypes of respiratory syncytial virus (RSV) in Bulgaria, 2016‐2018. J Med Virol. 2021;93:3401–11. https://doi.org/10.1002/jmv.26415

76. Klem N, Skjerven HO, Nilsen B, Brekke M, Vallersnes OM. Treatment for acute bronchiolitis before and after implementation of new national guidelines: a retrospective observational study from primary and secondary care in Oslo, Norway. BMJ Paediatr Open. 2021;5:e001111. https://doi.org/10.1136/bmjpo-2021-001111

77. Jensen A, Stensballe LG. Viral pneumonia in Danish children. Dan Med J. 2021.

78. Ippolito G, La Vecchia A, Umbrello G, Di Pietro G, Bono P, Scalia Catenacci S, et al. Disappearance of Seasonal Respiratory Viruses in Children Under Two Years Old During COVID-19 Pandemic: A Monocentric Retrospective Study in Milan, Italy. Front Pediatr. 2021;9. https://doi.org/10.3389/fped.2021.721005

79. Eklundh A, Rhedin S, Ryd-Rinder M, Andersson M, Gantelius J, Gaudenzi G, et al. Etiology of Clinical Community-Acquired Pneumonia in Swedish Children Aged 1–59 Months with High Pneumococcal Vaccine Coverage—The TREND Study. Vaccines (Basel). 2021;9:384. https://doi.org/10.3390/vaccines9040384

80. Drăgănescu AC, Miron VD, Streinu-Cercel A, Florea D, Vlaicu O, Bilaşco A, et al. Circulation of influenza A viruses among patients hospitalized for severe acute respiratory infection in a tertiary care hospital in Romania in the 2018/19 season. Medicine. 2021;100:e28460. https://doi.org/10.1097/MD.0000000000028460

81. Cannesson A, Elenga N. Community-Acquired Pneumonia Requiring Hospitalization among French Guianese Children. Int J Pediatr. 2021;2021:1–9. https://doi.org/10.1155/2021/4358818

82. Sobkowiak P, Mikoś M, Bręborowicz A, Szczepankiewicz A. Human bocavirus and metapneumovirus in acute wheezing in children—Is there a link with atopy? Clin Respir J. 2020;14:1201–7. https://doi.org/10.1111/crj.13261

83. Schneider UV, Holm MKA, Bang D, Petersen RF, Mortensen S, Trebbien R, et al. Point-of-care tests for influenza A and B viruses and RSV in emergency departments – indications, impact on patient management and possible gains by syndromic respiratory testing, Capital Region, Denmark, 2018. Eurosurveillance. 2020;25. https://doi.org/10.2807/1560-7917.ES.2020.25.44.1900430

84. Petrarca L, Nenna R, Frassanito A, Di Mattia G, Arima S, Pierangeli A, et al. Clustering Approach Identifies Different Profiles in Infants Hospitalized for Bronchiolitis. D24 LUNG INFECTION. American Thoracic Society; 2020. p. A6332–A6332. https://doi.org/10.1164/ajrccm-conference.2020.201.1_MeetingAbstracts.A6332

85. Papan C, Willersinn M, Weiß C, Karremann M, Schroten H, Tenenbaum T. Antibiotic utilization in hospitalized children under 2 years of age with influenza or respiratory syncytial virus infection – a comparative, retrospective analysis. BMC Infect Dis. 2020;20:606. https://doi.org/10.1186/s12879-020-05336-5

86. Nenna R, Frassanito A, Petrarca L, Di Mattia G, Midulla F. Age Limit in Bronchiolitis Diagnosis: 6 or 12 Months? Front Pediatr. 2020;8. https://doi.org/10.3389/fped.2020.00144

87. Comte A, Bour J-B, Darniot M, Pitoiset C, Aho-Glélé LS, Manoha C. Epidemiological characteristics and clinical outcomes of human rhinovirus infections in a hospitalized population. Severity is independently linked to RSV coinfection and comorbidities. Journal of Clinical Virology. 2020;125:104290. https://doi.org/10.1016/j.jcv.2020.104290

88. Calvo C, Alcolea S, Casas I, Pozo F, Iglesias M, Gonzalez-Esguevillas M, et al. A 14-year Prospective Study of Human Coronavirus Infections in Hospitalized Children. Pediatric Infectious Disease Journal. 2020;39:653–7. https://doi.org/10.1097/INF.0000000000002760

89. Bozzola E, Ciarlitto C, Guolo S, Brusco C, Cerone G, Antilici L, et al. Respiratory Syncytial Virus Bronchiolitis in Infancy: The Acute Hospitalization Cost. Front Pediatr. 2021;8. https://doi.org/10.3389/fped.2020.594898

90. Verbeke V, Reynders M, Floré K, Vandewal W, Debulpaep S, Sauer K, et al. Human bocavirus infection in Belgian children with respiratory tract disease. Arch Virol. 2019;164:2919–30. https://doi.org/10.1007/s00705-019-04396-6

91. Tsergouli K, Pappa S, Haidopoulou K, Gogou M, Giannopoulos A, Papa A. Respiratory Syncytial Virus in Greece, 2016–2018. Intervirology. 2019;62:210–5. https://doi.org/10.1159/000506049

92. Tramuto F, Restivo V, Costantino C, Colomba GME, Maida CM, Casuccio A, et al. Surveillance Data for Eight Consecutive Influenza Seasons in Sicily, Italy. Am J Trop Med Hyg. 2019;101:1232–9. https://doi.org/10.4269/ajtmh.19-0059

93. Szczawińska-Popłonyk A, Komasińska P, Tąpolska-Jóźwiak K, Więckowska B, Bręborowicz A. RSV versus non-RSV bronchiolitis in infants and young children – the bedside characteristics of one epidemic season. Pediatr Pol. 2019;94:18–24. https://doi.org/10.5114/polp.2019.83738

94. Souty C, Masse S, Valette M, Behillil S, Bonmarin I, Pino C, et al. Baseline characteristics and clinical symptoms related to respiratory viruses identified among patients presenting with influenza-like illness in primary care. Clinical Microbiology and Infection. 2019;25:1147–53. https://doi.org/10.1016/j.cmi.2019.01.014

95. Montes M, Oñate E, Muguruza A, Tamayo E, Carrera IM, Iturzaeta A, et al. Enterovirus D68 Causing Acute Respiratory Infection: Clinical Characteristics and Differences With Acute Respiratory Infections Associated With Enterovirus Non-D68. Pediatric Infectious Disease Journal. 2019;38:687–91. https://doi.org/10.1097/INF.0000000000002289

96. Ljubin-Sternak S, Meštrović T, Ivković-Jureković I, Tešović G, Mlinarić-Galinović G, Lukšić I, et al. High Detection Rates of Human Bocavirus in Infants and Small Children with Lower Respiratory Tract Infection from Croatia. Clin Lab. 2019;65. https://doi.org/10.7754/Clin.Lab.2018.180702

97. Korsun N, Angelova S, Trifonova I, Georgieva I, Voleva S, Tzotcheva I, et al. Viral pathogens associated with acute lower respiratory tract infections in children younger than 5 years of age in Bulgaria. Brazilian Journal of Microbiology. 2019;50:117–25. https://doi.org/10.1007/s42770-018-0033-2

98. Jagusic M, Slovic A, Ivancic-Jelecki J, Ljubin-Sternak S, Vilibić-Čavlek T, Tabain I, et al. Molecular epidemiology of human respiratory syncytial virus and human metapneumovirus in hospitalized children with acute respiratory infections in Croatia, 2014–2017. Infection, Genetics and Evolution. 2019;76:104039. https://doi.org/10.1016/j.meegid.2019.104039

99. Gaymard A, Phicone M, Ibranosyan M, Barthelemy D, Semanas Q, Simon B, et al. Epidemiology of respiratory syncytial virus circulating in Lyon, France, between 2014 and 2018. XXIe Journées Francophones de Virologie. 2019.

100. De Conto F, Conversano F, Medici MC, Ferraglia F, Pinardi F, Arcangeletti MC, et al. Epidemiology of human respiratory viruses in children with acute respiratory tract infection in a 3-year hospital-based survey in Northern Italy. Diagn Microbiol Infect Dis. 2019;94:260–7. https://doi.org/10.1016/j.diagmicrobio.2019.01.008

101. Cattoir L, Vankeerberghen A, Boel A, Van Vaerenbergh K, De Beenhouwer H. Epidemiology of RSV and hMPV in Belgium: a 10-year follow-up. Acta Clin Belg. 2019;74:229–35. https://doi.org/10.1080/17843286.2018.1492509

102. Barlotta A, Pirillo P, Stocchero M, Donato F, Giordano G, Bont L, et al. Metabolomic Profiling of Infants With Recurrent Wheezing After Bronchiolitis. J Infect Dis. 2019;219:1216–23. https://doi.org/10.1093/infdis/jiy659

103. Tsagarakis NJ, Sideri A, Makridis P, Triantafyllou A, Stamoulakatou A, Papadogeorgaki E. Age-related prevalence of common upper respiratory pathogens, based on the application of the FilmArray Respiratory panel in a tertiary hospital in Greece. Medicine. 2018;97:e10903. https://doi.org/10.1097/MD.0000000000010903

104. Sadasivam K, Venkataraman A. Abstract P-226: MENINGITIS IN BRONCHIOLITIS – HOW COMMON IS IT? Pediatric Critical Care Medicine. 2018;19:116–116. https://doi.org/10.1097/01.pcc.0000537683.99807.b0

105. Praznik A, Avinsek N, Prodan A, Grosek S, Erculj V, Pokorn M. Risk factors for severe bronchiolitis: A retrospective review of patients admitted to the university hospital in central region of slovenia. Eur J Pediatr. 2017. p. 1445–558. https://doi.org/10.1007/s00431-017-2979-8

106. Petrarca L, Nenna R, Frassanito A, Pierangeli A, Leonardi S, Scagnolari C, et al. Acute bronchiolitis: Influence of viral co‐infection in infants hospitalized over 12 consecutive epidemic seasons. J Med Virol. 2018;90:631–8. https://doi.org/10.1002/jmv.24994

107. Kurskaya O, Ryabichenko T, Leonova N, Shi W, Bi H, Sharshov K, et al. Viral etiology of acute respiratory infections in hospitalized children in Novosibirsk City, Russia (2013 – 2017). PLoS One. 2018;13:e0200117. https://doi.org/10.1371/journal.pone.0200117

108. Gil J, Almeida S, Constant C, Pinto S, Barreto R, Melo Cristino J, et al. Short-term relevance of lower respiratory viral coinfection in inpatients under 2 years of age. Anales de Pediatría (English Edition). 2018;88:127–35. https://doi.org/10.1016/j.anpede.2017.03.006

109. Ghazaly M, Thwaites R, Feather K, Ito K, RapePort G, Openshaw P, et al. G37(P) Laboratory correlates of severity in acute bronchiolitis of infancy. British Association for Paediatric Nephrology and Paediatric Intensive Care Society. BMJ Publishing Group Ltd and Royal College of Paediatrics and Child Health; 2018. p. A15.1-A15. https://doi.org/10.1136/archdischild-2018-rcpch.35

110. Ghazaly M, Nadel S. Characteristics of children admitted to intensive care with acute bronchiolitis. Eur J Pediatr. 2018;177:913–20. https://doi.org/10.1007/s00431-018-3138-6

111. Fillatre A, François C, Segard C, Duverlie G, Hecquet D, Pannier C, et al. Epidemiology and seasonality of acute respiratory infections in hospitalized children over four consecutive years (2012–2016). Journal of Clinical Virology. 2018;102:27–31. https://doi.org/10.1016/j.jcv.2018.02.010

112. Drazdienė N, Tamelienė R, Kviluna D, Saik P, Saik E, Zaikauskienė J. Hospitalisation of late preterm infants due to lower respiratory tract infections in Lithuania, Latvia, and Estonia: incidence, disease severity, and risk factors. Acta Med Litu. 2018;25:76–85. https://doi.org/10.6001/actamedica.v25i2.3760

113. Chakhunashvili G, Wagner AL, Power LE, Janusz CB, Machablishvili A, Karseladze I, et al. Severe Acute Respiratory Infection (SARI) sentinel surveillance in the country of Georgia, 2015-2017. PLoS One. 2018;13:e0201497. https://doi.org/10.1371/journal.pone.0201497

114. Botti C, Micillo A, Ricci G, Russo A, Denisco A, Cantile M, et al. Characterization of respiratory infection viruses in hospitalized children from Naples province in Southern Italy. Exp Ther Med. 2018; https://doi.org/10.3892/etm.2018.6061

115. Visseaux B, Collin G, Ichou H, Charpentier C, Bendhafer S, Dumitrescu M, et al. Usefulness of multiplex PCR methods and respiratory viruses’ distribution in children below 15 years old according to age, seasons and clinical units in France: A 3 years retrospective study. PLoS One. 2017;12:e0172809. https://doi.org/10.1371/journal.pone.0172809

116. Praznik A, Vinšek N, Prodan A, Erčulj V, Pokorn M, Mrvič T, et al. Risk factors for bronchiolitis severity: A retrospective review of patients admitted to the university hospital from central region of Slovenia. Influenza Other Respir Viruses. 2018;12:765–71. https://doi.org/10.1111/irv.12587

117. Paul SP, Mukherjee A, McAllister T, Harvey MJ, Clayton BA, Turner PC. Respiratory-syncytial-virus- and rhinovirus-related bronchiolitis in children aged &lt;2 years in an English district general hospital. Journal of Hospital Infection. 2017;96:360–5. https://doi.org/10.1016/j.jhin.2017.04.023

118. Nenna R, Evangelisti M, Frassanito A, Scagnolari C, Pierangeli A, Antonelli G, et al. Respiratory syncytial virus bronchiolitis, weather conditions and air pollution in an Italian urban area: An observational study. Environ Res. 2017;158:188–93. https://doi.org/10.1016/j.envres.2017.06.014

119. Moe N, Stenseng IH, Krokstad S, Christensen A, Skanke LH, Risnes KR, et al. The Burden of Human Metapneumovirus and Respiratory Syncytial Virus Infections in Hospitalized Norwegian Children. J Infect Dis. 2017;216:110–6. https://doi.org/10.1093/infdis/jix262

120. Moe N, Krokstad S, Stenseng IH, Christensen A, Skanke LH, Risnes KR, et al. Comparing Human Metapneumovirus and Respiratory Syncytial Virus: Viral Co-Detections, Genotypes and Risk Factors for Severe Disease. PLoS One. 2017;12:e0170200. https://doi.org/10.1371/journal.pone.0170200

121. Milić P, Sikirica M, Krželj V, Markić J. Characteristics of infants hospitalized  with bronchiolitis at University Hospital  of Split between 2011 and 2015. Paediatr Croat. 2017;61:8.

122. Leite S, Lachado A, Correia-Costa L, Fernandes A, Ramos A, Reis MG. Viral bronchiolitis and risk factors for severe outcome. Pediatr Pulmonol. 2017. https://doi.org/10.1002/ppul.23731

123. Korsun N, Angelova S, Tzotcheva I, Georgieva I, Lazova S, Parina S, et al. Prevalence and genetic characterisation of respiratory syncytial viruses circulating in Bulgaria during the 2014/15 and 2015/16 winter seasons. Pathog Glob Health. 2017;111:351–61. https://doi.org/10.1080/20477724.2017.1375708

124. García-García ML, Calvo C, Moreira A, Cañas JA, Pozo F, Sastre B, et al. Thymic stromal lymphopoietin, IL-33, and periostin in hospitalized infants with viral bronchiolitis. Medicine. 2017;96:e6787. https://doi.org/10.1097/MD.0000000000006787

125. Flores-González JC, Mayordomo-Colunga J, Jordan I, Miras-Veiga A, Montero-Valladares C, Olmedilla-Jodar M, et al. Prospective Multicentre Study on the Epidemiology and Current Therapeutic Management of Severe Bronchiolitis in Spain. Biomed Res Int. 2017;2017:1–7. https://doi.org/10.1155/2017/2565397

126. Chakhunashvili G, Wagner AL, Machablishvili A, Karseladze I, Tarkhan-Mouravi O, Zakhashvili K, et al. Implementation of a sentinel surveillance system for influenza-like illness (ILI) and severe acute respiratory infection (SARI) in the country of Georgia, 2015-2016. International Journal of Infectious Diseases. 2017;65:98–100. https://doi.org/10.1016/j.ijid.2017.09.028

127. Ampah P, Lane S, Stephenson S, Davis J, Van Meirt C, Flanagan B, et al. Respiratory morbidity after hospitalisation with Respiratory Syncytial Virus and Rhinovirus infection. Paediatric Respiratory Infection and Immunology. European Respiratory Society; 2017. p. OA4631. https://doi.org/10.1183/1393003.congress-2017.OA4631

128. Ahmed M, Kader S, Bandi S, Tang J. G371(P) Correlating virology and severity of bronchiolitis: use of bronchiolitis severity index. British Association of General Paediatrics and Paediatric Education Special Interest Group. BMJ Publishing Group Ltd and Royal College of Paediatrics and Child Health; 2017. p. A146.1-A146. https://doi.org/10.1136/archdischild-2017-313087.364

129. Voermans JJC, Seven-Deniz S, Fraaij PLA, van der Eijk AA, Koopmans MPG, Pas SD. Performance evaluation of a rapid molecular diagnostic, MultiCode based, sample-to-answer assay for the simultaneous detection of Influenza A, B and respiratory syncytial viruses. Journal of Clinical Virology. 2016;85:65–70. https://doi.org/10.1016/j.jcv.2016.10.019

130. Ugonna K, Douros K, Bingle CD, Everard ML. Cytokine responses in primary and secondary respiratory syncytial virus infections. Pediatr Res. 2016;79:946–50. https://doi.org/10.1038/pr.2016.29

131. Straňák Z, Saliba E, Kosma P, Posfay-Barbe K, Yunis K, Farstad T, et al. Predictors of RSV LRTI Hospitalization in Infants Born at 33 to 35 Weeks Gestational Age: A Large Multinational Study (PONI). PLoS One. 2016;11:e0157446. https://doi.org/10.1371/journal.pone.0157446

132. Richter J, Panayiotou C, Tryfonos C, Koptides D, Koliou M, Kalogirou N, et al. Aetiology of Acute Respiratory Tract Infections in Hospitalised Children in Cyprus. PLoS One. 2016;11:e0147041. https://doi.org/10.1371/journal.pone.0147041

133. Petrarca L, Nenna R, Frassanito A, Pierangeli A, Di Mattia G, Scagnolari C, et al. Human bocavirus in children hospitalized for acute respiratory tract infection in Rome. World Journal of Pediatrics. 2020;16:293–8. https://doi.org/10.1007/s12519-019-00324-5

134. Oliveira-Santos M, Santos JA, Soares J, Dias A, Quaresma M. Influence of meteorological conditions on RSV infection in Portugal. Int J Biometeorol. 2016;60:1807–17. https://doi.org/10.1007/s00484-016-1168-1

135. Ljubin-Sternak S, Marijan T, Ivković-Jureković I, Čepin-Bogović J, Gagro A, Vraneš J. Etiology and Clinical Characteristics of Single and Multiple Respiratory Virus Infections Diagnosed in Croatian Children in Two Respiratory Seasons. J Pathog. 2016;2016:1–8. https://doi.org/10.1155/2016/2168780

136. Gioula G, Melidou A, Exindari M, Papalexis P, Xanthis D, Malisiovas N. The burden of influenza, respiratory syncytial virus and human metapneumovirus in infants and young children in N. Greece, 2004-2013. Acta Medica International. 2016;3:154. https://doi.org/10.5530/ami.2016.1.32

137. Esposito S, Scarselli E, Lelii M, Scala A, Vitelli A, Capone S, et al. Antibody response to respiratory syncytial virus infection in children &lt;18 months old. Hum Vaccin Immunother. 2016;1–7. https://doi.org/10.1080/21645515.2016.1145847

138. Carrion T, Aliaga F, Ruiz M. Respiratory viruses in neonates. The Journal of Maternal-Fetal & Neonatal Medicine. 2016;29:1–313. https://doi.org/10.1080/14767058.2016.1191212

139. Cangiano G, Nenna R, Frassanito A, Evangelisti M, Nicolai A, Scagnolari C, et al. Bronchiolitis: Analysis of 10 consecutive epidemic seasons. Pediatr Pulmonol. 2016;51:1330–5. https://doi.org/10.1002/ppul.23476

140. Calvo C, García‐García ML, Pozo F, Carballo D, Martínez‐Monteserín E, Casas I. Infections and coinfections by respiratory human bocavirus during eight seasons in hospitalized children. J Med Virol. 2016;88:2052–8. https://doi.org/10.1002/jmv.24562

141. Berg AS, Inchley CS, Aase A, Fjaerli HO, Bull R, Aaberge I, et al. Etiology of Pneumonia in a Pediatric Population with High Pneumococcal Vaccine Coverage. Pediatric Infectious Disease Journal. 2016;35:e69–75. https://doi.org/10.1097/INF.0000000000001009

142. Beckmann C, Hirsch HH. Comparing Luminex NxTAG-Respiratory Pathogen Panel and RespiFinder-22 for multiplex detection of respiratory pathogens. J Med Virol. 2016;88:1319–24. https://doi.org/10.1002/jmv.24492

143. Armero G, Launes C, Hernández-Platero L, Alejandre C, Muñoz-Almagro C, Jordan I. Severe respiratory disease with rhinovirus detection: Role of bacteria in the most severe cases. Journal of Infection. 2016;73:506–9. https://doi.org/10.1016/j.jinf.2016.07.010

144. Tunsjø HS, Berg AS, Inchley CS, Røberg IK, Leegaard TM. Comparison of nasopharyngeal aspirate with flocked swab for PCR-detection of respiratory viruses in children. APMIS. 2015;123:473–7. https://doi.org/10.1111/apm.12375

145. Rhedin S, Lindstrand A, Hjelmgren A, Ryd-Rinder M, Öhrmalm L, Tolfvenstam T, et al. Respiratory viruses associated with community-acquired pneumonia in children: matched case–control study. Thorax. 2015;70:847–53. https://doi.org/10.1136/thoraxjnl-2015-206933

146. Cebey-López M, Herberg J, Pardo-Seco J, Gómez-Carballa A, Martinón-Torres N, Salas A, et al. Viral Co-Infections in Pediatric Patients Hospitalized with Lower Tract Acute Respiratory Infections. PLoS One. 2015;10:e0136526. https://doi.org/10.1371/journal.pone.0136526

147. Bakalovic G, Dzinovic A, Baljic R, Dizdar S, Selimovic A. Epidemiological Features of Bronchiolitis in the Pediatric Clinic of Clinical center of Sarajevo University. Materia Socio Medica. 2015;27:154. https://doi.org/10.5455/msm.2015.27.154-157

148. Aakula M, Bergroth E, Korppi M, Remes S, Piedra PA, Camargo CJA. Early predictors of asthma in children hospitalized for acute bronchiolitis: 4-year follow up. Allergy. 2015;70:1–106. https://doi.org/10.1111/all.12715

149. Tabatabai J, Prifert C, Pfeil J, Grulich-Henn J, Schnitzler P. Novel Respiratory Syncytial Virus (RSV) Genotype ON1 Predominates in Germany during Winter Season 2012–13. PLoS One. 2014;9:e109191. https://doi.org/10.1371/journal.pone.0109191

150. Sullivan C, Morgan C. PC.74 The importance of testing for Respiratory Syncytial Virus (RSV) in infants presenting with bronchiolitis who are receiving palivizumab. Arch Dis Child Fetal Neonatal Ed. 2014;99:A61.3-A61. https://doi.org/10.1136/archdischild-2014-306576.175

151. Skjerven HO, Megremis S, Carlsen K-H, Mowinckel P, Papadopoulos N, Lødrup Carlsen KC. Virus load and disease severity in acute bronchiolitis. 2014.

152. Serrano Lopez L, Jimenez Cabanillas V, Zamorano Bonilla L, Martin Alvarez E, Pena Caballero M, Hurtado Suazo JA. Incidence and determinants of respiratory syncitial virus bronchiolitis in late preterm infants. The Journal of Maternal-Fetal & Neonatal Medicine. 2014;27:1–437. https://doi.org/10.3109/14767058.2014.924236

153. Bekhof J, Wessels M, Reimink R, Bruijnesteijn L, Ruijs G, Brand P. Roomsharing in hospitalized children with bronchiolitis. European Respiratory Journal. 2014;44.

154. Zdanowicz K, Lewandowski D, Majewski P, Półkośnik K, Liwoch-Nienartowicz N, Reszeć-Giełażyn J, et al. Clinical Presentation and Co-Detection of Respiratory Pathogens in Children Under 5 Years with Non-COVID-19 Bacterial and Viral Respiratory Tract Infections: A Prospective Study in Białystok, Poland (2021-2022). Medical Science Monitor. 2023;29. https://doi.org/10.12659/MSM.941785

155. Williams TC, Marlow R, Hardelid P, Lyttle MD, Lewis KM, Mpamhanga CD, et al. The clinical impact of serious respiratory disease in children under the age of two during the 2021-2022 bronchiolitis season in the United Kingdom and Ireland. medRxiv. 2023;

156. Riepl A, Straßmayr L, Voitl P, Ehlmaier P, Voitl JJM, Langer K, et al. The surge of RSV and other respiratory viruses among children during the second COVID-19 pandemic winter season. Front Pediatr. 2023;11. https://doi.org/10.3389/fped.2023.1112150

157. Pungertnik T, Paro-Panjan D, Krivec U, Lozar Krivec J. The Diagnostic Value of Lung Ultrasound in Neonates and Infants with Acute Bronchiolitis. Central European Journal of Paediatrics. 2023;19:127. https://doi.org/10.5457/p2005-114.347

158. Nunziata F, Salomone S, Catzola A, Poeta M, Pagano F, Punzi L, et al. Clinical Presentation and Severity of SARS-CoV-2 Infection Compared to Respiratory Syncytial Virus and Other Viral Respiratory Infections in Children Less than Two Years of Age. Viruses. 2023;15:717. https://doi.org/10.3390/v15030717

159. Faraguna MC, Lepri I, Clavenna A, Bonati M, Vimercati C, Sala D, et al. The bronchiolitis epidemic in 2021–2022 during the SARS-CoV-2 pandemic: experience of a third level centre in Northern Italy. Ital J Pediatr. 2023;49:26. https://doi.org/10.1186/s13052-023-01425-8

160. Doenhardt M, Armann JP, Diffloth N, Gano C, Schneider J, Schneider DT, et al. High burden of acute respiratory tract infections leading to hospitalization at German pediatric hospitals: fall/winter 2022–2023. Infection. 2024;52:525–34. https://doi.org/10.1007/s15010-023-02123-7

161. De Rose DU, Maddaloni C, Martini L, Ronci S, Pugnaloni F, Marrocco G, et al. Are lung ultrasound features more severe in infants with bronchiolitis and coinfections? Front Pediatr. 2023;11. https://doi.org/10.3389/fped.2023.1238522

162. Curatola A, Graglia B, Ferretti S, Covino M, Pansini V, Eftimiadi G, et al. The acute bronchiolitis rebound in children after COVID-19 restrictions: a retrospective, observational analysis. Acta Biomed. 2023;94.

163. Castagno E, Raffaldi I, Del Monte F, Garazzino S, Bondone C. New epidemiological trends of respiratory syncytial virus bronchiolitis during COVID-19 pandemic. World Journal of Pediatrics. 2023;19:502–4. https://doi.org/10.1007/s12519-022-00623-4

164. Carlone G, Graziano G, Trotta D, Cafagno C, Aricò MO, Campodipietro G, et al. Bronchiolitis 2021–2022 epidemic: multicentric analysis of the characteristics and treatment approach in 214 children from different areas in Italy. Eur J Pediatr. 2023;182:1921–7. https://doi.org/10.1007/s00431-023-04853-0

165. Bravo-Queipo-de-Llano B, Sánchez García L, Casas I, Pozo F, La Banda L, Alcolea S, et al. Surveillance of Viral Respiratory Infections in the Neonatal Intensive Care Unit—Evolution in the Last 5 Years. Pathogens. 2023;12:644. https://doi.org/10.3390/pathogens12050644

166. Bardsley M, Morbey RA, Hughes HE, Beck CR, Watson CH, Zhao H, et al. Epidemiology of respiratory syncytial virus in children younger than 5 years in England during the COVID-19 pandemic, measured by laboratory, clinical, and syndromic surveillance: a retrospective observational study. Lancet Infect Dis. 2023;23:56–66. https://doi.org/10.1016/S1473-3099(22)00525-4

167. Baldassarre ME, Loconsole D, Centrone F, Caselli D, Martire B, Quartulli L, et al. Hospitalization for bronchiolitis in children aged ≤ 1year, Southern Italy, year 2021: need for new preventive strategies? Ital J Pediatr. 2023;49:66. https://doi.org/10.1186/s13052-023-01455-2

168. Balas WM, Śliwczyński A, Olszewski P, Gołębiak I, Sybilski AJ. Comparative Analysis of Symptomatology in Hospitalized Children with RSV, COVID-19, and Influenza Infections. Medical Science Monitor. 2023;29. https://doi.org/10.12659/MSM.941229

169. Aricò MO, Wrona D, Lavezzo G, Valletta E. Nasal CPAP in the Pediatric Ward to Reduce PICU Admissions for Severe Bronchiolitis? Pediatr Rep. 2023;15:599–607. https://doi.org/10.3390/pediatric15040055

170. Almeida T, Guimarães JT, Rebelo S. Epidemiological Changes in Respiratory Viral Infections in Children: The Influence of the COVID-19 Pandemic. Viruses. 2023;15:1880. https://doi.org/10.3390/v15091880

171. Vaux S, Viriot D, Forgeot C, Pontais I, Savitch Y, Barondeau-Leuret A, et al. Bronchiolitis epidemics in France during the SARS-CoV-2 pandemic: The 2020–2021 and 2021–2022 seasons. Infect Dis Now. 2022;52:374–8. https://doi.org/10.1016/j.idnow.2022.06.003

172. Roland D, Williams T, Lyttle MD, Marlow R, Hardelid P, Sinha I, et al. Features of the transposed seasonality of the 2021 RSV epidemic in the UK and Ireland: analysis of the first 10 000 patients. Arch Dis Child. 2022;107:1062–3. https://doi.org/10.1136/archdischild-2022-324241

173. Pappa S, Haidopoulou K, Zarras C, Theodorakou E, Papadimitriou E, Iosifidis E, et al. Early initiation of the respiratory syncytial virus season in 2021–2022, Greece. J Med Virol. 2022;94:3453–6. https://doi.org/10.1002/jmv.27671

174. Matera L, Nenna R, Frassanito A, Conti MG, Di Mattia G, Petrarca L, et al. RSV circulation during SARS-CoV-2 pandemic era: what happened in Italy. 0704 - Paediatric respiratory infection and immun. European Respiratory Society; 2022. p. 4138. https://doi.org/10.1183/13993003.congress-2022.4138

175. Maglione M, Pascarella A, Botti C, Ricci G, Morelli F, Camelia F, et al. Changing Epidemiology of Acute Viral Respiratory Infections in Hospitalized Children: The Post-Lockdown Effect. Children. 2022;9:1242. https://doi.org/10.3390/children9081242

176. Guitart C, Bobillo-Perez S, Alejandre C, Armero G, Launes C, Cambra FJ, et al. Bronchiolitis, epidemiological changes during the SARS-CoV-2 pandemic. BMC Infect Dis. 2022;22:84. https://doi.org/10.1186/s12879-022-07041-x

177. Flores-Pérez P, Gerig N, Cabrera-López MI, de Unzueta-Roch JL, del Rosal T, Calvo C. Acute bronchiolitis during the COVID-19 pandemic. Enferm Infecc Microbiol Clin. 2022;40:572–5. https://doi.org/10.1016/j.eimc.2021.06.012

178. Duclos M, Hommel B, Allantaz F, Powell M, Posteraro B, Sanguinetti M. Multiplex PCR Detection of Respiratory Tract Infections in SARS-CoV-2-Negative Patients Admitted to the Emergency Department: an International Multicenter Study during the COVID-19 Pandemic. Microbiol Spectr. 2022;10. https://doi.org/10.1128/spectrum.02368-22

179. Cozzi G, Cortellazzo Wiel L, Amaddeo A, Gatto A, Giangreco M, Klein-Kremer A. Supplementum 258: Abstracts of the annual meeting 2022 of the Swiss Society of Paediatrics. Swiss Med Wkly. 2022;152:w30203. https://doi.org/10.4414/SMW.2022.w30203

180. Cerar S, Pirnovar V. A Comparative Analysis of the Occurrence of Lower Respiratory Tract Infections Caused by Respiratory Syncytial Virus among Newborns in the Years before and during Covid-19 Pandemic at a Tertiary Referral Hospital in Slovenia. Central European Journal of Paediatrics. 2022;18:100. https://doi.org/10.5457/p2005-114.322

181. Cason C, Zamagni G, Cozzi G, Tonegutto D, Ronfani L, Oretti C, et al. Spread of Respiratory Pathogens During the COVID-19 Pandemic Among Children in the Northeast of Italy. Front Microbiol. 2022;13. https://doi.org/10.3389/fmicb.2022.804700

182. Camporesi A, Morello R, Ferro V, Pierantoni L, Rocca A, Lanari M, et al. Epidemiology, Microbiology and Severity of Bronchiolitis in the First Post-Lockdown Cold Season in Three Different Geographical Areas in Italy: A Prospective, Observational Study. Children. 2022;9:491. https://doi.org/10.3390/children9040491

183. Buonsenso D, Morello R, Ferro V, Musolino A, De Rose C, Inchingolo R, et al. Are Lung Ultrasound Features More Severe in Children Diagnosed with Bronchiolitis after the COVID-19 Lockdown Period? J Clin Med. 2022;11:5294. https://doi.org/10.3390/jcm11185294

184. Diesner-Treiber SC, Voitl P, Voitl JJM, Langer K, Kuzio U, Riepl A, et al. Respiratory Infections in Children During a Covid-19 Pandemic Winter. Front Pediatr. 2021;9. https://doi.org/10.3389/fped.2021.740785

185. Coyle N, F. Guinness F, Fitzsimons J. Respiratory admissions during COVID-19 pandemic restrictions-a single centre experience. Cogent Med. 2021;8. https://doi.org/10.1080/2331205X.2021.2002558

186. Calderaro A, De Conto F, Buttrini M, Piccolo G, Montecchini S, Maccari C, et al. Human respiratory viruses, including SARS-CoV-2, circulating in the winter season 2019–2020 in Parma, Northern Italy. International Journal of Infectious Diseases. 2021;102:79–84. https://doi.org/10.1016/j.ijid.2020.09.1473

187. Vittucci AC, Piccioni L, Coltella L, Ciarlitto C, Antilici L, Bozzola E, et al. The Disappearance of Respiratory Viruses in Children during the COVID-19 Pandemic. Int J Environ Res Public Health. 2021;18:9550. https://doi.org/10.3390/ijerph18189550

188. Mattila J, Thomas E, Lehtinen P, Vuorinen T, Waris M, Heikkinen T. Burden of influenza during the first year of life. Influenza Other Respir Viruses. 2021;15:506–12. https://doi.org/10.1111/irv.12820

189. Bakalovic G, Dzinovic A, Baljic R, Dizdar S, Selimovic A. Epidemiological Features of Bronchiolitis in the Pediatric Clinic of Clinical center of Sarajevo University. Materia Socio Medica. 2015;27:154. https://doi.org/10.5455/msm.2015.27.154-157

190. Straňák Z, Saliba E, Kosma P, Posfay-Barbe K, Yunis K, Farstad T, et al. Predictors of RSV LRTI Hospitalization in Infants Born at 33 to 35 Weeks Gestational Age: A Large Multinational Study (PONI). PLoS One. 2016;11:e0157446. https://doi.org/10.1371/journal.pone.0157446

191. Flores-González JC, Mayordomo-Colunga J, Jordan I, Miras-Veiga A, Montero-Valladares C, Olmedilla-Jodar M, et al. Prospective Multicentre Study on the Epidemiology and Current Therapeutic Management of Severe Bronchiolitis in Spain. Biomed Res Int. 2017;2017:1–7. https://doi.org/10.1155/2017/2565397

192. García-García ML, Calvo C, Moreira A, Cañas JA, Pozo F, Sastre B, et al. Thymic stromal lymphopoietin, IL-33, and periostin in hospitalized infants with viral bronchiolitis. Medicine. 2017;96:e6787. https://doi.org/10.1097/MD.0000000000006787

193. Drazdienė N, Tamelienė R, Kviluna D, Saik P, Saik E, Zaikauskienė J. Hospitalisation of late preterm infants due to lower respiratory tract infections in Lithuania, Latvia, and Estonia: incidence, disease severity, and risk factors. Acta Med Litu. 2018;25:76–85. https://doi.org/10.6001/actamedica.v25i2.3760

194. Drăgănescu AC, Miron VD, Streinu-Cercel A, Florea D, Vlaicu O, Bilaşco A, et al. Circulation of influenza A viruses among patients hospitalized for severe acute respiratory infection in a tertiary care hospital in Romania in the 2018/19 season. Medicine. 2021;100:e28460. https://doi.org/10.1097/MD.0000000000028460

195. Oh D-Y, Biere B, Grenz M, Wolff T, Schweiger B, Dürrwald R, et al. Virological Surveillance and Molecular Characterization of Human Parainfluenzavirus Infection in Children with Acute Respiratory Illness: Germany, 2015–2019. Microorganisms. 2021;9:1508. https://doi.org/10.3390/microorganisms9071508

196. Penela-Sánchez D, González-de-Audicana J, Armero G, Henares D, Esteva C, de-Sevilla M-F, et al. Lower Respiratory Tract Infection and Genus Enterovirus in Children Requiring Intensive Care: Clinical Manifestations and Impact of Viral Co-Infections. Viruses. 2021;13:2059. https://doi.org/10.3390/v13102059

197. Petat H, Gajdos V, Angoulvant F, Vidalain P-O, Corbet S, Marguet C, et al. High Frequency of Viral Co-Detections in Acute Bronchiolitis. Viruses. 2021;13:990. https://doi.org/10.3390/v13060990

198. Sominina A, Danilenko D, Komissarov A, Pisareva M, Musaeva T, Bakaev M, et al. Age-Specific Etiology of Severe Acute Respiratory Infections and Influenza Vaccine Effectivity in Prevention of Hospitalization in Russia, 2018–2019 Season. J Epidemiol Glob Health. 2021;11:413–25. https://doi.org/10.1007/s44197-021-00009-1

199. Kohns Vasconcelos M, Loens K, Sigfrid L, Iosifidis E, Epalza C, Donà D, et al. Aetiology of acute respiratory infection in preschool children requiring hospitalisation in Europe—results from the PED-MERMAIDS multicentre case–control study. BMJ Open Respir Res. 2021;8:e000887. https://doi.org/10.1136/bmjresp-2021-000887

200. Rudan I, O’Brien KL, Nair H, Liu L, Theodoratou E, Qazi S, et al. Epidemiology and etiology of childhood pneumonia in 2010: Estimates of incidence, severe morbidity, mortality, underlying risk factors and causative pathogens for 192 countries. J Glob Health. University of Edinburgh; 2013;3. https://doi.org/10.7189/jogh.03.010401

201. Pratt MTG, Abdalla T, Richmond PC, Moore HC, Snelling TL, Blyth CC, et al. Prevalence of respiratory viruses in community-acquired pneumonia in children: a systematic review and meta-analysis. Lancet Child Adolesc Health. 2022;6:555–70. https://doi.org/10.1016/S2352-4642(22)00092-X

202. Kenmoe S, Kengne-Nde C, Ebogo-Belobo JT, Mbaga DS, Fatawou Modiyinji A, Njouom R. Systematic review and meta-analysis of the prevalence of common respiratory viruses in children &lt; 2 years with bronchiolitis in the pre-COVID-19 pandemic era. PLoS One. 2020;15:e0242302. https://doi.org/10.1371/journal.pone.0242302

203. Midulla F, Scagnolari C, Bonci E, Pierangeli A, Antonelli G, De Angelis D, et al. Respiratory syncytial virus, human bocavirus and rhinovirus bronchiolitis in infants. Arch Dis Child. 2010;95:35–41. https://doi.org/10.1136/adc.2008.153361

204. Van Benten I, Koopman L, Niesters B, Hop W, Van Middelkoop B, De Waal L, et al. Predominance of rhinovirus in the nose of symptomatic and asymptomatic infants. Pediatric Allergy and Immunology. 2003;14:363–70. https://doi.org/10.1034/j.1399-3038.2003.00064.x

205. Wang H, Zheng Y, Deng J, Wang W, Liu P, Yang F, et al. Prevalence of respiratory viruses among children hospitalized from respiratory infections in Shenzhen, China. Virol J. 2016;13:39. https://doi.org/10.1186/s12985-016-0493-7

206. Hasegawa K, Linnemann RW, Avadhanula V, Mansbach JM, Piedra PA, Gern JE, et al. Detection of respiratory syncytial virus and rhinovirus in healthy infants. BMC Res Notes. 2015;8:718. https://doi.org/10.1186/s13104-015-1695-6

207. Kumar P, Medigeshi GR, Mishra VS, Islam M, Randev S, Mukherjee A, et al. Etiology of Acute Respiratory Infections in Infants. Pediatric Infectious Disease Journal. 2017;36:25–30. https://doi.org/10.1097/INF.0000000000001359

208. Tapia MD, Sylla M, Driscoll AJ, Touré A, Kourouma N, Sissoko S, et al. The Etiology of Childhood Pneumonia in Mali. Pediatric Infectious Disease Journal. 2021;40:S18–28. https://doi.org/10.1097/INF.0000000000002767

209. Gastaldi A, Donà D, Barbieri E, Giaquinto C, Bont LJ, Baraldi E. COVID-19 Lesson for Respiratory Syncytial Virus (RSV): Hygiene Works. Children. 2021;8:1144. https://doi.org/10.3390/children8121144

210. Chung Ong K. The Impact of Non-pharmacological Interventions in Reducing Hospitalizations for Non-COVID Respiratory Illnesses during COVID-19. Chronic Obstructive Pulmonary Disease - Contemporary and Consummate Management of a Systemic Disorder. IntechOpen; 2025. https://doi.org/10.5772/intechopen.1012640

211. Principi N, Autore G, Ramundo G, Esposito S. Epidemiology of Respiratory Infections during the COVID-19 Pandemic. Viruses. 2023;15:1160. https://doi.org/10.3390/v15051160

212. Chow EJ, Uyeki TM, Chu HY. The Effects of The COVID-19 Pandemic on Community Respiratory Virus Activity. Nat Rev Microbiol. 2022; https://doi.org/10.1038/s41579-022-00807-9

213. Kampf G. Efficacy of Ethanol against Viruses in Hand Disinfection. J Hosp Infect. 2018;98:331–8. https://doi.org/10.1016/j.jhin.2017.08.025

214. Boone SA, Gerba CP. Significance of Fomites in the Spread of Respiratory and Enteric Viral Disease. Appl Environ Microbiol. 2007;73:1687–96. https://doi.org/10.1128/AEM.02051-06

215. Treggiari D, Piubelli C, Formenti F, Silva R, Perandin F. Resurgence of Respiratory Virus after Relaxation of COVID-19 Containment Measures: A Real-World Data Study from a Regional Hospital of Italy. Int J Microbiol. 2022;2022:1–5. https://doi.org/10.1155/2022/4915678

216. Jia R, Lu L, Su L, Lin Z, Gao D, Lv H, et al. Resurgence of Respiratory Syncytial Virus Infection During COVID-19 Pandemic Among Children in Shanghai, China. Front Microbiol. 2022;13. https://doi.org/10.3389/fmicb.2022.938372

217. Hodjat P, Christensen PA, Subedi S, Bernard DW, Olsen RJ, Long SW. The Reemergence of Seasonal Respiratory Viruses in Houston, Texas, after Relaxing COVID-19 Restrictions. Microbiol Spectr. 2021;9. https://doi.org/10.1128/Spectrum.00430-21

218. Billard M-N, Bont LJ. Quantifying the RSV Immunity Debt Following COVID-19: A Public Health Matter. Lancet Infect Dis. 2023;23:3–5. https://doi.org/10.1016/S1473-3099(22)00544-8

219. Shi T, Huang L, Tian J. Epidemiology of Respiratory Infections during the Different Levels of Non-pharmaceutical Interventions. BMC Pediatr. 2025;25:375. https://doi.org/10.1186/s12887-025-05723-4

220. Abu-Raya B, Viñeta Paramo M, Reicherz F, Lavoie PM. Why Has the Epidemiology of RSV Changed during the COVID-19 Pandemic? EClinicalMedicine. 2023;61:102089. https://doi.org/10.1016/j.eclinm.2023.102089

221. Grobben M, Juncker HG, van der Straten K, Lavell AHA, Schinkel M, Buis DTP, et al. Decreased Passive Immunity to Respiratory Viruses through Human Milk during the COVID-19 Pandemic. Microbiol Spectr. 2022;10. https://doi.org/10.1128/spectrum.00405-22

222. Sullivan SG, Carlson S, Cheng AC, Chilver MB, Dwyer DE, Irwin M, et al. Where Has All the Influenza Gone? The Impact of COVID-19 on the Circulation of Influenza and Other Respiratory Viruses, Australia, March to September 2020. Eurosurveillance. 2020;25. https://doi.org/10.2807/1560-7917.ES.2020.25.47.2001847

223. Baker RE, Park SW, Yang W, Vecchi GA, Metcalf CJE, Grenfell BT. The Impact of COVID-19 Nonpharmaceutical Interventions on the Future Dynamics of Endemic Infections. Proc Natl Acad Sci U S A. 2020;117:30547–53. https://doi.org/10.1073/pnas.2013182117

224. Huang QS, Wood T, Jelley L, Jennings T, Jefferies S, Daniells K, et al. Impact of the COVID-19 Nonpharmaceutical Interventions on Influenza and Other Respiratory Viral Infections in New Zealand. Nat Commun. 2021;12:1001. https://doi.org/10.1038/s41467-021-21157-9

225. Piret J, Boivin G. Viral Interference between Respiratory Viruses. Emerg Infect Dis. 2022;28:273–81. https://doi.org/10.3201/eid2802.211727

226. Hall CB, Weinberg GA, Iwane MK, Blumkin AK, Edwards KM, Poehling KA, et al. The Burden of Respiratory Syncytial Virus Infection in Young Children. N Engl J Med. 2009;360:588–98.

227. Jacobs SE, Lamson DM, St. George K, Walsh TJ. Human Rhinoviruses. Clin Microbiol Rev. 2013;26:135–62. https://doi.org/10.1128/CMR.00077-12

228. Nickbakhsh S, Mair C, Matthews L, Reeve R, Johnson PCD, Thorburn F, et al. Virus–virus Interactions Impact the Population Dynamics of Influenza and the Common Cold. Proc Natl Acad Sci USA. 2019;116:27142–50. https://doi.org/10.1073/pnas.1911083116

229. Wu A, Mihaylova VT, Landry ML, Foxman EF. Interference between Rhinovirus and Influenza A Virus: A Clinical Data Analysis and Experimental Infection Study. Lancet Microbe. 2020;1:e254–62. https://doi.org/10.1016/S2666-5247(20)30114-2

230. Yeoh DK, Foley DA, Minney-Smith CA, Martin AC, Mace AO, Sikazwe CT, et al. Impact of Coronavirus Disease 2019 Public Health Measures on Detections of Influenza and Respiratory Syncytial Virus in Children During the 2020 Australian Winter. Clin Infect Dis. 2021;72:2199–202. https://doi.org/10.1093/cid/ciaa1475

231. Kissler SM, Tedijanto C, Goldstein E, Grad YH, Lipsitch M. Projecting the Transmission Dynamics of SARS-CoV-2 through the Postpandemic Period. Science (1979). 2020;368:860–8. https://doi.org/10.1126/science.abb5793
